# Effect of active warming on perioperative cardiovascular outcomes: a systematic review and meta-analysis of randomized controlled trials

**DOI:** 10.1007/s00540-023-03205-4

**Published:** 2023-06-08

**Authors:** Yunying Feng, Yuelun Zhang, Boyuan Sun, Yumiao He, Lijian Pei, Yuguang Huang

**Affiliations:** 1 Department of Anesthesiology, Peking Union Medical College Hospital, Chinese Academy of Medical Sciences and Peking Union Medical College, 1 Shuaifuyuan, Dongcheng District, Beijing, 100730 China; 2 Joint Laboratory of Anesthesia and Pain, Chinese Academy of Medical Sciences and Peking Union Medical College, Beijing, China; 3 Eight-Year Program of Clinical Medicine, Peking Union Medical College Hospital, Chinese Academy of Medical Sciences and Peking Union Medical College, Beijing, China; 4 Medical Research Centre, Peking Union Medical College Hospital, Chinese Academy of Medical Sciences and Peking Union Medical College, Beijing, China; 5 Clinical Epidemiology Unit, Peking Union Medical College Hospital, Chinese Academy of Medical Sciences and Peking Union Medical College, Beijing, China

**Keywords:** Hypothermia, Perioperative care, Cardiovascular events

## Abstract

**Purpose:**

The objective of this study was to provide an updated review on the active warming effects on major adverse cardiac events, 30-day all-cause mortality, and myocardial injury after noncardiac surgery.

**Method:**

We systematically searched MEDLINE, EMBASE, CINAHL, Cochrane CENTRAL, Web of Science, and Chinese BioMedical Literature Database. We included randomized controlled trials of adult population undergoing noncardiac surgeries that concentrate on the comparison of active warming methods and passive thermal management. Cochrane Collaboration’s tool was applied for risk-of-bias assessment. We used trial sequential analysis to evaluate the possibility of false positive or negative results.

**Results:**

A total of 13,316 unique records were identified, of which only 19 with reported perioperative cardiovascular outcomes were included in the systematic review and nine of them were included in final meta-analysis. No statistically significant difference between active warming methods and routine care was found in major adverse cardiac events (RR 0.56, 95% confidence interval (CI) 0.14–2.21, *I*^2^ = 71%, number of events 59 vs. 70), 30-day all-cause mortality (RR 0.81, 95% CI 0.43–1.54, *I*^2^ = 0%, number of events 17 vs. 21), and myocardial injury after noncardiac surgery (RR 0.61, 95% CI 0.17–2.22, *I*^2^ = 79%, number of events 236 vs. 234). Trial sequential analysis suggests that current trials did not reach the minimum information size regarding the major cardiovascular events.

**Conclusions:**

Compared to routine perioperative care, we found that active warming methods are not necessary for cardiovascular prevention in patients undergoing noncardiac surgery.

**Supplementary Information:**

The online version contains supplementary material available at 10.1007/s00540-023-03205-4.

## Introduction

Every year, more than 4.2 million patients die within the first 30 days after surgery, making postoperative deaths the third greatest contributor to global death [1]. Among patients undergoing non-cardiac surgery, about one-third of postoperative deaths are associated with major adverse cardiac events (MACE) [2]. And a growing number of studies indicate that more surgical patients suffered from myocardial injury after non-cardiac surgery (MINS) without signs and symptoms [3–5]. These perioperative cardiovascular outcomes could result in severe surgical adverse events, prolonged hospitalization, and increased medical costs, challenging perioperative care.

One possible factor explaining cardiovascular outcomes is inadvertent perioperative hypothermia (IPH) [6]. IPH is defined as a central body temperature lower than 36.0 °C [7]. It is a common adverse effect of surgery with a reported prevalence ranging from 50 to 90% [8]. Presumed mechanisms of IPH-relevant cardiovascular outcomes include thermoregulatory vasoconstriction causing blood pressure increase [9], sympathetic activation leading to tachycardia [10], and worsen oxygen supply in demand ischemia [11]. Although the extent of IPH’s contribution to perioperative cardiovascular risk remains unclear, aggressive interventions to maintain body temperature have been explored for reducing postoperative myocardial injury and cardiac morbidity.

Active warming methods, including electric blankets, warm-water mattresses, intravenous fluids warming, and anesthetic air warming, could transfer extra heat to the surgical patients, thus compensate the heat loss [12]. Previous meta-analyses of randomized controlled trials (RCTs) have shown their application for preventing perioperative shivering, surgical-site infection, and blood loss [13–15]. For cardiovascular outcomes and all-cause mortality, however, limited data were analyzed based on low-quality evidence. Current conclusions of active warming on postoperative cardiovascular risk reduction were mainly drawn from an RCT of 300 patients with high coronary artery risk [16]. Yet, the cardiac events in this trial were assessed based on 48-h electrocardiogram monitoring, which was insensitive and would miss most asymptomatic (no chest pain or other symptoms) myocardial injuries. Given that more relevant RCTs [17, 18], especially the PROTECT trial [19], have been published since the latest meta-analysis [15], an updated analysis of IPH and active warming methods on myocardial injury as well as cardiac mortality is necessary. Given the large sample size in the PROTECT trial, whether further trials need to be conducted in this field also should be evaluated. Therefore, this study aimed to provide an up-to-date overview of the effects of active warming methods on MACE and all-cause mortality after non-cardiac surgeries; gain more reliable estimates of IPH’s role in perioperative cardiac complications; and conduct the trial sequential analysis (TSA) to quantify the possibility of false negative findings.

## Methods

This systematic review was reported following the Preferred Reporting Items for Systematic reviews and Meta-analyses (PRISMA) 2020 statement [20]. The protocol of this study was registered on PROSPERO (CRD42022332368, 3 August 2022).

### Eligibility criteria

We included RCTs of adult population undergoing non-cardiac surgeries that focus on the comparison of active warming methods and passive thermal management. Active warming systems to prevent unintended hypothermia include electric blankets, heated mattresses, forced-air warmers, and warmed and humidified carbon dioxide. Passive thermal management includes warmed cotton baskets and other thermal insulations. Intravenous fluids warming is supposed to be a type of active warming methods according to traditional definition, and has been part of routine care in some medical institutions. However, it cannot compensate for the core-to-periphery redistribution of body heat, which is usually the initial effect of anesthesia and leads to redistribution hypothermia [12]. Therefore, we included RCTs investigating intravenous fluids warming on perioperative cardiovascular complications. If the control group in RCT was given routine care with fluids warming, it would be included as well. Both active and passive thermoregulatory interventions should be used preoperatively or intraoperatively. We excluded RCTs where interventions were only applied postoperatively because these applications are usually related to intentional hypothermia.

### Information source and search strategy

We developed search strategy for the following major public electronic biomedicine databases: Ovid MEDLINE, EMBASE, CINAHL (EBSCO), Cochrane CENTRAL, Web of Science, and Chinese BioMedical Literature Database. The search range of publication time was from inception to 23 May 2022 (see Supplementary Table 1 in Appendix 1). Reference lists of previous systematic reviews and meta-analyses on this topic were also checked to find more relevant studies. Potentially relevant abstracts and preprints were searched in Google Scholar. Additionally, we searched ClinicalTrials.gov for ongoing trials and adverse event reports of completed trials. No restrictions were set on language.

### Selection process

Publications identified through database searching were imported into Endnote 20.1 software (Thomson Reuters, Toronto, Ontario, Canada). After deduplication, two authors (YF, BS) independently screened titles and abstracts of the citations in initial screening, and further the full texts of potentially eligible citations in the final screening. Disagreements in both initial screening and final screening were solved by discussion with other reviewers in our team. The excluded citations during the screening were listed and noted in the PRISMA flow diagram.

### Data collection and risk-of-bias assessment

Using a pilot data collection form, two authors (YF, BS) extracted data from finally included studies separately and verified the results. Detailed information of thermal management type, covered locations, surgical type, and anesthesia type was collected. The primary outcomes of the study were MACE (i.e., a composite of cardiovascular death, cardiac arrest, cardiogenic shock, and hemodynamically significant complete heart block) reported within 30 days after non-cardiac surgery, and 30-day all-cause mortality. The secondary outcomes included MINS, other cardiovascular complications (e.g., perioperative hypotension, arrhythmia), duration of ICU or post-anesthesia care unit (PACU) stay, and hospital stay. Conflicts were resolved through discussion or by consulting other members of the review team. We assessed publication bias by investigating the funnel plot symmetry.

Two authors (YF, BS) independently assessed the methodological quality of the included studies according to the Cochrane Collaboration’s tool for assessing risk of bias [21]. Domains of selection bias, performance bias, detection bias, attrition bias, and reporting bias were graded at high, low, or unclear risk. Disagreements were resolved by a third author (YZ).

### Statistical analysis

Eligible studies were first evaluated from clinical and methodologic perspectives to check their homogeneity. If no obvious heterogeneity exists, perioperative cardiovascular complications will be pooled as binary outcomes using RR as effect measure. Random-effects model with restricted maximum-likelihood estimator for between-study variance would be used to combine eligible trials, and the tau-squared (tau^2^) was used for estimating the relation between study variances. Forest plots were used to present the results, depicted using Review Manager 5 (RevMan 5.4.1).

TSA is a recently developed cumulative meta-analysis method for weighing type I and type II errors as well as estimating when the effect is large enough to be unaffected by further studies [22]. We examined the possibility of false positive or negative findings of current evidence by TSA version 0.9 (Copenhagen Trial Unit, Copenhagen, Denmark). We mainly focused on the primary outcomes, which are major perioperative outcomes that should be taken seriously. MINS was also analyzed. An overall 5% risk of a type I error and a power of 80% were maintained for TSA. We calculated the information size required to detect or reject a minimal relevant difference of incidence of perioperative cardiovascular complications. The trial sequential monitoring boundaries (TSMBs) were calculated using the Lan-DeMets version [23] of the O’Brien–Fleming function [24].

## Results

### Search and screening

**Fig. 1:**
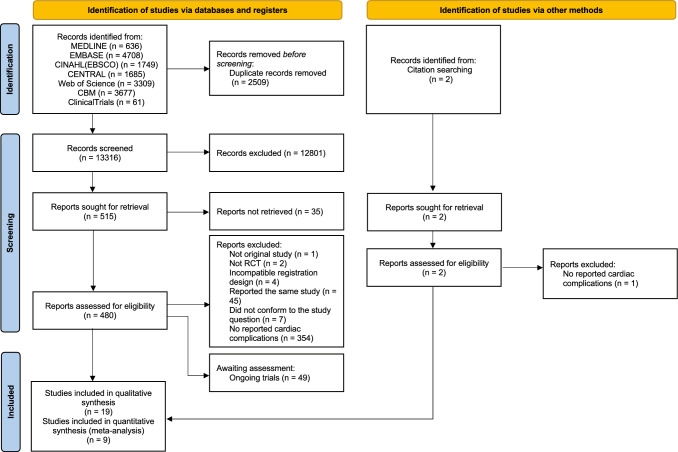
Study flow diagram

Applying the strategy shown in Supplementary Table 1 (Appendix 1), the electronic search was conducted on 23 May 2022 in Ovid MEDLINE, EMBASE, CINAHL (EBSCO), Cochrane CENTRAL, Web of Science, and Chinese BioMedical Literature Database. A total of 15,825 publications were identified with 2509 duplicates (Fig. 1). After screening 13,316 titles and abstracts, 515 studies remained for further evaluation according to the predefined eligibility criteria. Nineteen studies were included in qualitative synthesis after screening the full texts, while 9 studies were finally included in meta-analysis. Information of the full-text screened publications is listed in Appendix 2.

### Characteristics of included studies

The included studies were conducted in various non-cardiac surgeries applying different anesthesia types (Supplementary Table 2, Appendix 3). Most of the patients were given general anesthesia, while other patients undergoing hip arthroplasty or cesarean delivery were given spinal anesthesia [25–28]. Seventeen studies were focused on the forced-air warming application for preventing perioperative hypothermia and relevant complications, other perioperative thermal interventions were warming mattress, circulating-water mattress, and warm and humidified insufflation gas. Patients divided into the control group usually received no active warming, or only routine care with intravenous fluids warming. Seventeen studies applied interventions in the active warming group during intra-operative phase, while six of them added pre-operative active warming methods to prewarm the patients for a certain hour. Apart from one large multicenter study [19], most eligible studies recruited 30–300 patients in the research. A few studies reported the primary outcomes focused in our review.

### Risk of bias assessment

**Fig. 2:**
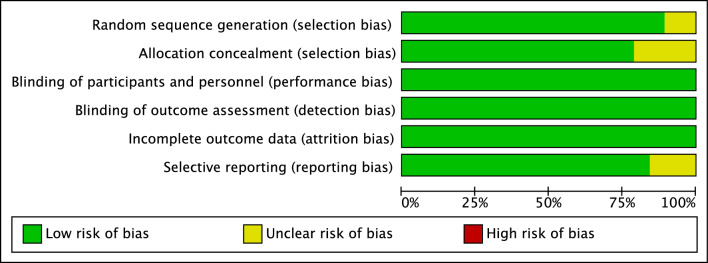
Risk of bias graph

Risk of bias judgements is provided in Fig. 2 and Supplementary Fig. 1 (Appendix 3). Some studies failed to report sufficient information to allow a judgment to be made for random sequence generation or allocation, so it is unclear whether selection bias was present in these studies. Blinding participants or investigators to the warming intervention was generally impractical, yet the objective indices and obvious clinical events in this study were unlikely to be affected by detection bias. Exclusions from study analyses were reported in the included studies in this review. No serious issues with attrition were identified. Several studies did not report sufficient results because of incomplete follow-up, but the direction of this effect is unclear. Regarding publication bias, funnel plots are shown in Supplementary Fig. 2 (Appendix 3).

### Perioperative cardiovascular complications

**Fig. 3:**
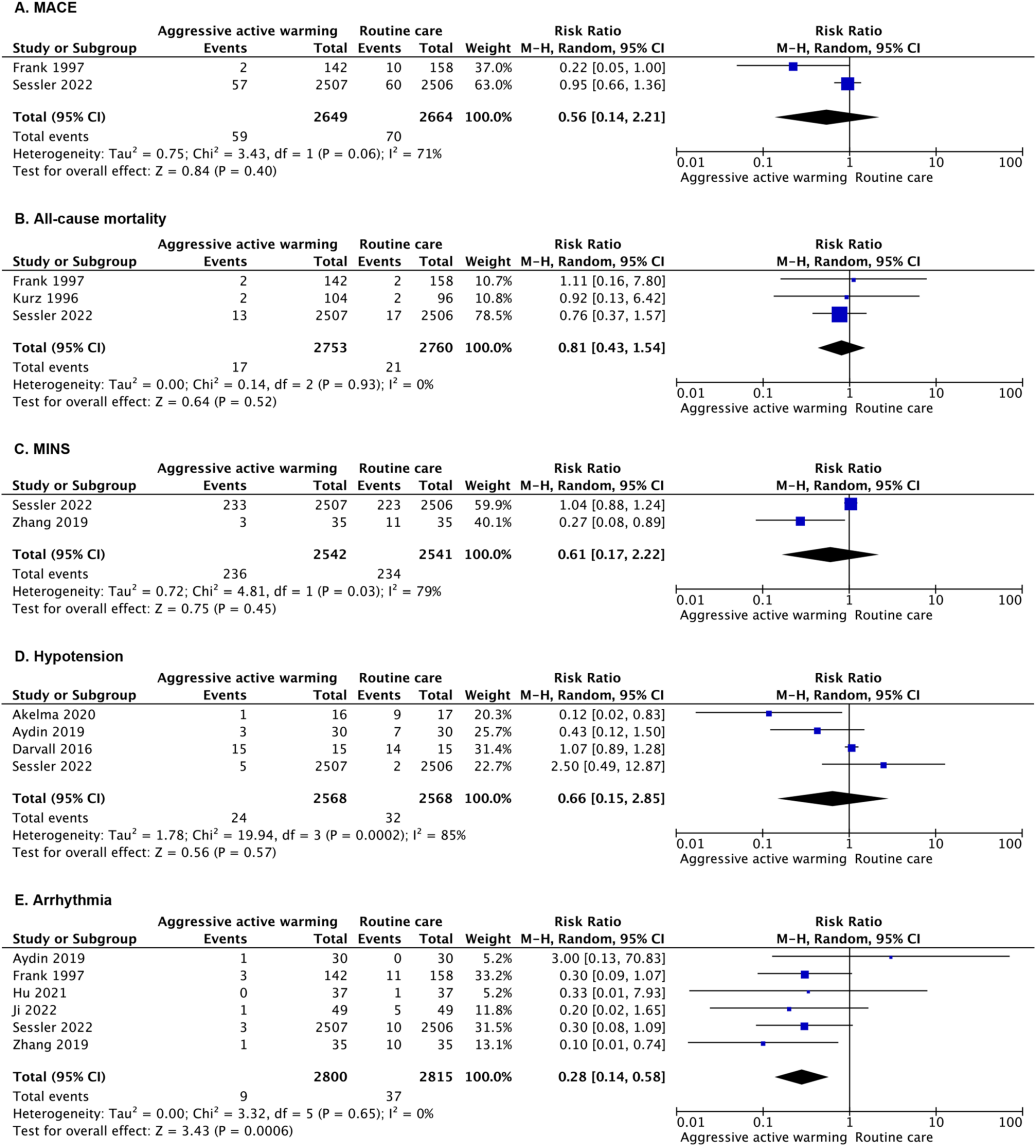
Meta-analyses of perioperative cardiovascular complications. CI indicates confidence interval. **A** meta-analysis of major adverse cardiac events (MACE); **B** meta-analysis of 30-day all-cause mortality; **C** meta-analysis of myocardial injury after non-cardiac surgery (MINS); **D** meta-analysis of perioperative hypotension; **E** meta-analysis of perioperative arrhythmia

Among all the included studies, nine of them had non-zero event records with comparable anesthesia type and active thermal interventions, available for data synthesis (Supplementary Table 3, Appendix 3). All of the nine studies reported higher mean core temperature measured at the end of surgery in the active groups. The data on perioperative complications were sparsely reported. Therefore, we conducted meta-analysis on forced-air warming comparing to passive warming or routine care only with intravenous fluids warming (Fig. 3).

Here are the results for primary outcomes. The two studies (5313 patients) [16, 19] that assessed the risk of MACE showed no significant differences between active warming and routine care (RR 0.56, 95% confidence interval (CI) 0.14–2.21, number of events 59 vs. 70). All-cause mortality within 30 days was assessed in three trials (5513 patients) [16, 19, 29], and no statistically significant difference in risk of 30-day all-cause mortality was noted when active warming was compared with routine care or no active warming (RR 0.81, 95% CI 0.43–1.54, number of events 17 vs. 21).

As for secondary outcomes, the two studies (5083 patients) [18, 19] that assessed the risk of MINS indicated no significant differences between active warming and routine care (RR 0.61, 95% CI 0.17–2.22, number of events 236 vs. 234). Results from four trials (5136 patients) [19, 30–32] showed no significant difference between active warming and passive insulation on perioperative hypotension (RR 0.66, 95% CI 0.15 to 2.85, number of events 24 vs 32). Perioperative arrhythmia was assessed in six trials (5615 patients) [16, 18, 19, 31, 33, 34]. The pooled estimate demonstrated a significant reduction of arrhythmia with active warming strategy (RR 0.28, 95% CI 0.14 to 0.58, number of events 9 vs 37). Since the results of PACU stay, ICU stay, and hospital stay were seldom reported and presented in different data forms, we did not combine these potential heterogeneous values. Due to limited eligible studies, we did not further conduct planned subgroup analyses.

### Trial sequential analysis

**Fig. 4:**
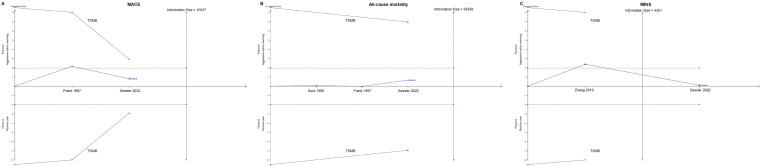
Trial sequential analysis (TSA) of the effects of active warming vs routine care on major perioperative cardiovascular outcomes. **A** TSA for major adverse cardiac events (MACE); **B** TSA for 30-day all-cause mortality; **C** TSA for myocardial injury after non-cardiac surgery (MINS)

Results of TSA analysis are shown in Fig. 4. For MACE, the required information size was 10,327 patients for 80% power and an overall 5% probability of a type I error (Fig. 4a). This number was much larger than the number of randomized patients in current three trials (5313 patients). The cumulative Z-curve crossed neither the traditional boundary nor the TSMB, suggesting a lack of firm evidence. Similarly, the cumulative Z-curve crossed neither the traditional boundary nor the TSMB for the outcome of 30-day all-cause mortality (Fig. 4b). For the outcome of MINS, the required information size was 4301, which was lower than the number of patients randomized in the two trials (Fig. 4c). Cumulative Z-curve did not cross the traditional boundary or the TSMB, indicating firm evidence for insignificant difference between active warming and routine care.

## Discussion

The results of our study provide an updated review on the effects of active warming methods on perioperative cardiovascular outcomes and all-cause mortality in non-cardiac surgeries. Our findings indicate that the application of active warming methods could reduce the risk of perioperative arrhythmia, yet is not associated with MACE, 30-day all-cause mortality, MINS, or perioperative hypotension. TSA suggests firm evidence for no risk reduction of MINS using active warming methods.

Currently, active warming methods are applied mainly on the skin surface, such as forced-air warming, circulating water, and resistive heating. Among them, forced-air warming has advantages in safety and convenience, and is the most commonly studied strategy [35]. Previous studies and meta-analyses have evaluated the efficacy of active warming methods on maintaining core body temperature, reducing blood transfusion, and preventing surgical-site infection and shivering [14, 15, 36]. The most recent meta-analysis evaluated one randomized trials that enrolled 300 patients for MACE, and two randomized trials that enrolled 500 patients for all-cause mortality, suggesting no significant differences with low-quality evidence. To fully evaluate the efficacy of active warming on perioperative cardiovascular outcomes, we summarized the updated data and only included non-zero event trials. Newly included trials involved more patients and more advanced techniques, such as troponin examination, for cardiovascular risk evaluation. Meta-analyses in our study reveal that active warming strategy had no significant difference with routine care in preventing MACE and 30-day all-cause mortality, in line with the latest meta-analysis. Findings may be due to bearable hemodynamic changes caused by mild thermal dysregulation for non-cardiac surgery patients in the short run, and positive inotropic and oxygen-sparing effects of hypothermia [37]. Instead, pre-existing cardiovascular risk factors would be more common to induce severe cardiovascular complications after surgical stress [38]. TSA in our study showed a lack of evidence for active warming efficacy of reducing risk for MACE and mortality. Given that these perioperative complications were relatively rare, further studies aiming at the active thermal management for preventing perioperative cardiovascular complications and mortality could hardly reach the required information size.

MINS is a newly established clinical diagnosis that describes the myocardial infarction and ischemic myocardial injury after non-cardiac surgery that do not fulfill the universal definition of myocardial infarction [39]. It is associated with mortality and major vascular complications [40]. Our results raised an important issue that active warming strategy reduce the risk of arrhythmia, yet had no protective effect on MINS. The evidence is further confirmed by TSA. As an important surrogate outcome for more clinically relevant cardiovascular events, MINS was not changed by active warming; hence, it is unlikely to reverse the current evidence even with large trials reporting MACE, all-cause mortality, and other cardiac outcomes in future. It seems that perioperative active thermal management is effective in reducing thermal discomfort, wound infection, and shivering, rather than preventing cardiovascular complications. Thus, we recommend further high-quality researches on active thermal strategy in non-cardiac surgery should concentrate on other perioperative outcomes instead of cardiovascular complications, and report any relevant MACE, all-cause mortality, MINS, and other cardiac outcomes in complication reports.

Our review has limitations. First, publication bias may be introduced for the outcomes collected in this review, yet this kind of bias is unable to be further assessed through funnel plots due to the small number of included studies. Second, the great clinical variability or heterogeneity in the identified trials hindered the further interpretation of the results. The considerable advances in perioperative managements and surgical techniques in the past decades made the synthesis even more challengeable. In relation to control groups, some studies applied passive insulation only, while others used routine care strategy including intravenous fluids warming. As for intervention group, aggressive active warming strategies were not explored for duration. We planned to conduct a network meta-analysis comparing all the warming strategies, but the limited number of eligible trials and sparse outcome events failed to support such analysis. Moreover, the number of final included RCTs in our meta-analysis is relatively small, and our results are mainly affected by one RCT with large population. Therefore, the results should be taken cautiously with those ethnic groups that were not included in the primary RCTs. Finally, patients’ core temperature in control group was usually kept between a narrow range for ethical reasons, so it is unknown whether the extent of hypothermia would be associated with perioperative cardiovascular outcomes.

## Conclusion

Perioperative cardiovascular outcomes and all-cause mortality did not differ significantly in patients receiving active warming strategy or routine care. Application of active warming methods is not necessary for cardiovascular complication prevention in non-cardiac surgery.

## Supplementary Information

Below is the link to the electronic supplementary material.

Supplementary file1 (DOCX 40 KB)

Supplementary file1 (DOCX 74 KB)

Supplementary file1 (DOCX 3479 KB)

## Data Availability

The datasets generated and/or analyzed during this study are available from the corresponding author on reasonable request.
