# Supplementary material for: Effect of active warming on perioperative cardiovascular outcomes: a systematic review and meta-analysis of randomized controlled trials
Source: J Anesth. 2023 Jun 8;37(4):573–81. doi: 10.1007/s00540-023-03205-4 (PMC10390383; doi:10.1007/s00540-023-03205-4)
Supplement: Supplementary file 1 — Supplementary file1 (DOCX 40 KB) [file 540_2023_3205_MOESM1_ESM.docx]

**Effect of active warming on perioperative cardiovascular outcomes: a systematic review and meta-analysis of randomized controlled trials**

**Journal of Anesthesia**

Yunying Feng, Yuelun Zhang, Boyuan Sun, Yumiao He, Lijian Pei, Yuguang Huang^*^

*Correspondence to: Prof. Yuguang Huang, M.D., Department of Anesthesiology, Peking Union Medical College Hospital, Chinese Academy of Medical Sciences and Peking Union Medical College, 1 Shuaifuyuan, Dongcheng District, 100730 Beijing, China (email: [garypumch@163.com](mailto:garypumch@163.com)).

Supplementary Table 1. Searched sources and search strategies.

Here we provide the search strategies applied in Ovid MEDLINE(R), EMBASE, CINAHL, Cochrane CENTRAL, Web of Science, and Chinese BioMedical Literature Database.

| Databases | Search terms | Amount |
| --- | --- | --- |
| Ovid MEDLINE(R)  Date of search: 23 May 2022 | 1 Heating/ or Body Temperature/ or Rewarming/  2 (Active warming system* or ((Mattress* or blanket*) adj3 (warm water or Electric)) or Forced-air warming or (((Intravenous or irrigation) adj3 fluid*) and warming) or (CO2 adj6 warming) or (an?esthetic adj6 warming) or ((thermal or temperature) adj3 manag*)).mp. or (warming or blanket*).ti,ab.  3 #1 OR #2  4 Surgical Procedures, Operative/ or General Surgery/ or exp Postoperative Complications/ or exp Intraoperative Complications/ or exp Perioperative Care/ or exp Postoperative Care/ or exp Preoperative Care/  5 ((operat* or recovery) adj3 room*).ti. or ((operat* or surg*) adj3 complic*).ab. or (surg* or operat*).ab. or (post?operativ* or pre?operativ* or peri?operativ*).ab.  6 #4 OR #5  7 #3 AND #6  8 (BairHugger or Bair Hugger or ThermaCare or Gaymar or Optisan or WarmAir or FilteredFlow or WarmTouch or CareDrape or Life-Air or Snuggle Warm or Warm-Gard).mp.  9 #7 OR #8  10 ((randomiserandomised controlled trial or controlled clinical trial).pt. or randomiserandomised.ab. or placebo.ab. or clinical trials as topic.sh. or randomly.ab. or trial.ti.) not (animals not (humans and animals)).sh.  11 #9 AND #10 | 636 |
| EMBASE  Date of search: 23 May 2022 | 1 'Active warming system*':ab,ti  2 ((Mattress* OR blanket*) NEAR/3 ('warm water' OR electric)):ab,ti  3 'Forced-air warming':ab,ti  4 (((Intravenous OR irrigation) NEAR/3 fluid*) AND 'warming'):ab,ti  5 (CO2 NEAR/6 warming):ab,ti  6 ((Thermal OR temperature) NEAR/3 manag*):ab,ti  7 Warming:ab,ti OR blanket*:ab,ti  8 #1 OR #2 OR #3 OR #4 OR #5 OR #6 OR #7  9 'Warming'/exp OR 'heating'/exp OR 'body temperature'/exp OR 'hypothermia'/exp  10 #8 OR #9  11 ((Operat* OR recovery) NEAR/3 room*):ab,ti  12 ((Operat* OR surg*) NEAR/3 complic*):ab,ti OR surg*:ab,ti OR operat*:ab,ti OR ((post?operativ* OR pre?operativ* OR peri?operativ*) NEAR/3 complicat*):ab,ti  13 'Surgery'/exp OR 'operating room'/exp OR 'recovery room'/exp OR 'perioperative complication'/exp OR 'postoperative complication'/exp OR 'preoperative care'/exp OR 'postoperative care'/exp  14 #11 OR #12 OR #13  15 #10 AND #14  16 'Bairhugger' OR 'bair hugger'/exp OR 'thermacare' OR 'gaymar' OR 'optisan' OR 'warmair' OR 'filteredflow' OR 'warmtouch'/exp OR 'caredrape' OR 'life air' OR 'snuggle warm' OR 'warm gard'  17 #15 OR #16  18 ((((Singl* OR doubl* OR tripl*) NEAR/3 blind) OR crossover:ab,ti OR multicenter:ab OR 'placebo'/de OR placebo OR controlled) AND study:ab OR random*:ab,ti OR trial*:ab,ti) NOT [animal cell]/lim NOT [animal experiment]/lim NOT [animal model]/lim NOT [animal tissue]/lim  19 #17 AND #18 | 4,708 |
| CINAHL (EBSCO)  Date of search: 23 May 2022 | 1 (MH "Body Temperature") or (MH "Core Body Temperature")  2 (MH "Heating")  3 (MH "Warming Techniques") or (MM "Hypothermia Treatment (Iowa NIC)")  4 TX Active warming system* or TX ( ((Mattress* or blanket*) and (warm water or Electric)) ) or TX Forced-air warming or TX ( (((Intravenous or irrigation) and fluid*) and warming) ) or TX CO2 N3 warming or TX an?esthetic N3 warming or TX ( ((thermal or temperature) and manag*) ) or TI (warming or blanket* ) or AB ( warming or blanket* )  5 S1 or S2 or S3 or S4  6 (MH "Surgery, Operative") or (MH "Operating Rooms")  7 (MH "Post Anesthesia Care Units") or (MH "Patients' Rooms")  8 TI ( (operat* or recovery) and room* ) or AB ( (operat* or recovery) and room* )  9 (MH "Postoperative Complications+")  10 AB ( post?operativ* or pre?operativ* or peri?operativ* ) or AB ( surg* or operat* ) or TI ( surg* or operat*) or TX ( (operat* or surg*) and complic* )  11 S6 or S7 or S8 or S9 or S10  12 S5 and S11  13 TX BairHugger OR Bair Hugger OR ThermaCare OR Gaymar OR Optisan OR WarmAir OR FilteredFlow OR WarmTouch OR CareDrape OR Life-Air OR Snuggle Warm OR Warm-Gard  14 S12 or S13  15 (MM "Random Assignment")  16 MH "Clinical Trials+"  17 (MM "Placebos") or (MM "Multicenter Studies") or (MM "Crossover Design")  18 (MM "Double-Blind Studies") or (MM "Single-Blind Studies") or (MM "Triple-Blind Studies")  19 AB random* or TI trail* or AB ( placebo* or mulicenter or crossover )  20 AB ( (double or single or triple) and (blind* or mask*) ) or TI ( (double or single or triple) and (blind* or mask*))  21 S15 or S16 or S17 or S18 or S19 or S20  22 S14 and S21 | 1,749 |
| Cochrane CENTRAL  Date of search: 23 May 2022 | 1 MeSH descriptor: [Body Temperature] this term only  2 MeSH descriptor: [Heating] this term only  3 MeSH descriptor: [Rewarming] explode all trees  4 ((Active warming system*) or ((Mattress* or blanket*) near (warm water or Electric)) or Forced-air warming or (((Intravenous or irrigation) near fluid*) and warming) or (CO2 near warming) or (an?esthetic near warming) or ((thermal or temperature) near manag*) or (warming or blanket*)):ti,ab,kw  5 #1 OR #2 OR #3 OR #4  6 MeSH descriptor: [Surgical Procedures, Operative] this term only  7 MeSH descriptor: [Operating Rooms] explode all trees  8 MeSH descriptor: [Recovery Room] explode all trees  9 ((operat* or recovery) near room*):ti,ab,kw  10 ((operat* or recovery) near room*):ti,ab,kw  11 MeSH descriptor: [Intraoperative Complications] explode all trees  12 MeSH descriptor: [Postoperative Complications] this term only  13 MeSH descriptor: [Preoperative Care] explode all trees  14 MeSH descriptor: [Postoperative Care] explode all trees  15 MeSH descriptor: [Intraoperative Care] explode all trees  16 ((operat* OR surg*) near complic*):ti,ab OR (surg* OR operat*):ti,ab OR (post?operativ* OR pre?operativ* OR peri?operativ*):ab  17 #6 OR #7 OR #8 OR #9 OR #10 OR #11 OR #12 OR #13 OR #14 OR #15 OR #16  18 #5 AND #17  19 BairHugger OR Bair Hugger OR ThermaCare OR Gaymar OR Optisan OR WarmAir OR FilteredFlow OR WarmTouch OR CareDrape OR Life-Air OR Snuggle Warm OR Warm-Gard  20 #18 OR #19 in Trials | 1,685 |
| Web of Science  Date of search: 23 May 2022 | 1 TS=((hypo?therm* or normo?therm* or thermo?regulat* or shiver*) or ((thermal or temperature) SAME (regulat* or manage* or maintain*)) or (low* SAME temperature*) or thermo?genesis or ((reduc* or prevent*) and temperature and (decrease or decline)) or (heat SAME (preserv* or loss or retention or retain* or balance)) or (core SAME (thermal or temperature*)))  2 TS=((intervention* SAME treat*) or (vasodilatat* or infrared light* or intravenous nutrient* or warming system*) or ((Mattress* or blanket*) SAME (warm water or Electric)) or (warm* and (air or CO2 or fluid* or an?esthetic* or IV or gas* or device* or patient* or passive* or active* or skin or surg*))) or TI=(warming or blanket*) or TI=(pharmacological agent* or thermal insulat* or pre?warm* or re?warm*)  3 #1 and #2  4 TS=(random* or (trial* SAME (control* or clinical*)) or placebo* or multicenter* or prospective* or ((blind* or mask*) SAME (single or double or triple or treble)))  5 #3 and #4 | 3,309 |
| Chinese BioMedical Literature Database  Date of search: 23 May 2022 | 1 (((((("体温变化"[不加权:扩展] OR "低温"[不加权:扩展]) OR "体温"[不加权:扩展]) OR "体温调节"[不加权:扩展]) OR "皮肤温度"[不加权:扩展])) OR "采暖"[不加权:扩展]) OR "复温"[不加权:扩展]  Key word explanation:  "体温变化" means “body temperature changes”  "低温" means “low temperature”  "体温" means “body temperature”  "体温调节" means “body temperature regulation”  "皮肤温度" means “skin temperature”  "采暖" means “heating”  "复温" means “rewarming”  [不加权:扩展] means expanding the search to include related terms and synonyms  2 "保温"[全部字段] OR "恒温"[全部字段] OR "升温"[全部字段] OR "加热"[全部字段] OR "电热毯"[全部字段] OR "隔热"[全部字段]  Key word explanation:  "保温" means “heat retention”  "恒温" means “constant temperature”  "加热" means “heating”  "电热毯" means “electric blanket”  "隔热" means “insulation”  [全部字段] means searching for these keywords and phrases across all fields in the database or search engine  3 (#1) OR (#2)  4 (((((("手术前护理"[不加权:扩展] OR "监测, 手术中"[不加权:扩展]) OR "围手术期护理"[不加权:扩展]) OR "手术室护理"[不加权:扩展]) OR "手术后并发症"[不加权:扩展]) OR "手术中并发症"[不加权:扩展]) OR "手术期间"[不加权:扩展]) OR "围手术期"[不加权:扩展]  Key word explanation:  "手术前护理" means “preoperative care”  "监测, 手术中" means “intraoperative monitoring”  "围手术期护理" means “perioperative care”  "手术室护理" means “management in operating room”  "手术后并发症" means “postoperative complications”  "手术中并发症" means “intraoperative complications”  "手术期间" means “during surgery”  "围手术期" means “perioperative period”  [不加权:扩展] means expanding the search to include related terms and synonyms  5 (#3) AND (#4)  6 ("随机对照试验"[不加权:扩展]) OR "临床对照试验"[不加权:扩展]  Key word explanation:  "随机对照试验" means “randomized controlled trial”  "临床对照试验" means “clinical controlled trial”  [不加权:扩展] means expanding the search to include related terms and synonyms  7 (("随机"[摘要] OR "试验"[摘要]) OR "分组"[摘要]) OR "对照"[摘要]  Key word explanation:  "随机" means “randomize”  "试验" means “trial”  "分组" means “grouping”  "对照" means “control”  [摘要] means limiting the search to the abstracts of articles or publications  8 (#6) OR (#7)  9 (#5) AND (#8) AND "随机对照试验"[文献类型]  Key word explanation:  "随机对照试验" means “randomized controlled trial”  [文献类型] means limiting the search to articles or publications of a specific document type  10 "动物"[特征词] NOT "人类"[特征词]  Key word explanation:  "动物" means “animal”  "人类" means “human”  [特征词] means specifying the keyword or subject  11 (#9) NOT (#10) | 3,677 |
| Total | | 15,764 |
| Total after de-duplication | | 13,286 |
