# Supplementary material for: Effect of active warming on perioperative cardiovascular outcomes: a systematic review and meta-analysis of randomized controlled trials
Source: J Anesth. 2023 Jun 8;37(4):573–81. doi: 10.1007/s00540-023-03205-4 (PMC10390383; doi:10.1007/s00540-023-03205-4)
Supplement: Supplementary file 2 — Supplementary file1 (DOCX 74 KB) [file 540_2023_3205_MOESM2_ESM.docx]

**Effect of active warming on perioperative cardiovascular outcomes: a systematic review and meta-analysis of randomized controlled trials**

**Journal of Anesthesia**

Yunying Feng, Yuelun Zhang, Boyuan Sun, Yumiao He, Lijian Pei, Yuguang Huang^*^

*Correspondence to: Prof. Yuguang Huang, M.D., Department of Anesthesiology, Peking Union Medical College Hospital, Chinese Academy of Medical Sciences and Peking Union Medical College, 1 Shuaifuyuan, Dongcheng District, 100730 Beijing, China (email: [garypumch@163.com](mailto:garypumch@163.com)).

**References to studies included in this review**

Aydin, H., Simsek, T., & Demiraran, Y. (2019). Effects of Inadvertent Perioperative Hypothermia on Metabolic and Inflammatory Mediators. TURKISH JOURNAL OF ANAESTHESIOLOGY AND REANIMATION, 47(6), 448-455.

Becerra, A., Valencia, L., Saavedra, P., Rodriguez-Perez, A., & Villar, J. (2021). Effect of prewarming on body temperature in short-term bladder or prostatic transurethral resection under general anesthesia: A randomized, double-blind, controlled trial [Randomized Controlled Trial Research Support, Non-U.S. Gov't]. Scientific Reports, 11(1), 20762.

Breuer, M., Wittenborn, J., Rossaint, R., Van Waesberghe, J., Kowark, A., Mathei, D., Keszei, A., Tchaikovski, S., Zeppernick, M., Zeppernick, F., Stickeler, E., Zoremba, N., Meinhold-Heerlein, I., & Bruells, C. (2022). Warm and humidified insufflation gas during gynecologic laparoscopic surgery reduces postoperative pain in predisposed patients—a randomized, controlled multi-arm trial [Article]. Surgical Endoscopy, 36(6), 4154-4170.

Canturk, M., Canturk, F. K., Kocaoglu, N., & Hakki, M. (2019). [The effects of crystalloid warming on maternal body temperature and fetal outcomes: a randomized controlled trial] [Randomized Controlled Trial]. Brazilian Journal of Anesthesiology, 69(1), 13-19. (Os efeitos do aquecimento de cristaloides sobre a temperatura corporal materna e nas condicoes fetais: ensaio clinico randomico)

Chiang, N., Rodda, O. A., Sleigh, J., & Vasudevan, T. (2017). Perioperative warming, oxygen, and Ilomedin on oxygenation and healing in infrainguinal bypass surgery [Article]. Journal of Surgical Research, 220, 197-205.

Darvall, J., Vijayakumar, R., & Leslie, K. (2016). Prewarming neurosurgical patients to minimize hypotension on induction of anesthesia: a randomized trial [Comparative Study Randomized Controlled Trial Research Support, Non-U.S. Gov't]. Canadian Journal of Anaesthesia, 63(5), 577-583.

Frank, S. M., Fleisher, L. A., Breslow, M. J., Higgins, M. S., Olson, K. F., Kelly, S., & Beattie, C. (1997). Perioperative maintenance of normothermia reduces the incidence of morbid cardiac events. A randomized clinical trial [Clinical Trial Randomized Controlled Trial Research Support, Non-U.S. Gov't Research Support, U.S. Gov't, P.H.S.]. JAMA, 277(14), 1127-1134.

Ji, J., Gu, X., & Xiao, C. (2022). Comparison of Perioperative Active or Routine Temperature Management on Postoperative Quality of Recovery in PACU in Patients Undergoing Thoracoscopic Lobectomy: a Randomized Controlled Study [Journal: Article]. International Journal of General Medicine, 15, 429‐436.

Kavak Akelma, F., Ergil, J., Özkan, D., Arık, E., Baran Akkuş, İ., & Aydın, G. B. (2020). The Effect of Preoperative Warming on Postoperative Hypothermia. Gulhane Medical Journal, 62(2), 114-120.

Kim, H. Y., Lee, K. C., Lee, M. J., Kim, M.-N., Kim, J.-S., Lee, W. S., & Lee, J. H. (2014). Comparison of the efficacy of a forced-air warming system and circulating-water mattress on core temperature and post-anesthesia shivering in elderly patients undergoing total knee arthroplasty under spinal anesthesia. Korean Journal of Anesthesiology, 66(5), 352-357.

Kurz, A., Sessler, D. I., & Lenhardt, R. (1996). Perioperative normothermia to reduce the incidence of surgical-wound infection and shorten hospitalization [Article]. New England Journal of Medicine, 334(19), 1209-1215.

NCT01626690. (2012). Prospective Trial of the Effect of Preoperative Forced-air Warming on Perioperative Body Temperature Following Neuraxial Anesthesia in Total Hip Arthroplasty Patients.

Ni, T. T., Zhou, Z. F., He, B., & Zhou, Q. H. (2020). Effects of combined warmed preoperative forced-air and warmed perioperative intravenous fluids on maternal temperature during cesarean section: a prospective, randomized, controlled clinical trial [Randomized Controlled Trial Research Support, Non-U.S. Gov't]. BMC Anesthesiology, 20(1), 48.

Sessler, D. I., Pei, L., Li, K., Cui, S., Chan, M. T. V., Huang, Y., Wu, J., He, X., Bajracharya, G. R., Rivas, E., & Lam, C. K. M. (2022). Aggressive intraoperative warming versus routine thermal management during non-cardiac surgery (PROTECT): a multicentre, parallel group, superiority trial [Article]. Lancet (London, England), 399(10337), 1799-1808.

Unlugenc Hakki, G. E., Ilgınel Murat, Unal Ilker, Emre, Mustafa. (2018). Does warming intravenous fluids during spinal-induced hypotension decrease the incidence of hypotension and reduce the amount of fluid, transfusion and ephedrine requirements? Middle East journal of anaesthesiology, 25.

Wang, J. Y., Fang, P., Sun, G. Q., & Li, M. (2022). Effect of active forced air warming during the first hour after anesthesia induction and intraoperation avoids hypothermia in elderly patients. BMC Anesthesiology, 22(1). https://doi.org/10.1186/s12871-022-01577-w

Wong, P. F., Kumar, S., Bohra, A., Whetter, D., & Leaper, D. J. (2007). Randomized clinical trial of perioperative systemic warming in major elective abdominal surgery [Randomized Controlled Trial]. British Journal of Surgery, 94(4), 421-426.

Zhang, Z., Xu, M., Wu, D., Zhang, X., & Wu, J. (2019). Postoperative Myocardial Injury in Middle-Aged and Elderly Patients Following Curative Resection of Esophageal Cancer With Aggressive or Standard Body Temperature Management: A Randomized Controlled Trial [Randomized Controlled Trial]. Anesthesia & Analgesia, 129(2), 352-359.

胡艳, & 马继. (2021). 充气式保温毯在结肠癌手术患者护理中的应用效果. 医疗装备, 34(18), 2.

**References to studies excluded from this review**

Actrn. (2016). Warm humidification for prevention of hypothermia during liver transplantation.

Ahn, H. Y., & Eom, M. R. (2010). Rewarming Intervention Program for Abdominal Surgery Patients. Journal of korean academy of fundamentals of nursing, 17(2), 220‐230.

Ahn, S. W., & Kim, T. H. (1999). The Effects of Warming Intravenous Fluids, Sensory Block Level, and Skin Temperature on Postanesthetic Shivering during Spinal Anesthesia. Korean Journal of Anesthesiology, 37(5), 787‐792.

Akhtar, Z., Hesler, B. D., Fiffick, A. N., Mascha, E. J., Sessler, D. I., Kurz, A., Ayad, S., & Drmed, L. S. (2016). A randomized trial of prewarming on patient satisfaction and thermal comfort in outpatient surgery. Journal of Clinical Anesthesia, 33, 376-385.

Aksu, C., İçli, A. D., Toker, K., & Solak, Z. M. (2017). The effects of irrigation solution temperature on body temperature in transurethral surgeries [Article]. Transüretral Cerrahide İrrigasyon Solüsyonunun Sıcaklığının Vücut Sıcaklığına Etkileri, 37(3), 109-113.

Alparslan, V., Kus, A., Hosten, T., Ertargin, M., Ozdamar, D., Toker, K., & Solak, M. (2018). Comparison of forced-air warming systems in prevention of intraoperative hypothermia [Article]. Journal of Clinical Monitoring and Computing, 32(2), 343-349.

Andrzejowski, J., Hoyle, J., Eapen, G., Turnbull, D., Andrzejowski, J., Hoyle, J., Eapen, G., & Turnbull, D. (2008). Effect of prewarming on post-induction core temperature and the incidence of inadvertent perioperative hypothermia in patients undergoing general anaesthesia. BJA: The British Journal of Anaesthesia, 101(5), 627-631.

Andrzejowski, J. C., Turnbull, D., Nandakumar, A., Gowthaman, S., & Eapen, G. (2010). A randomised single blinded study of the administration of pre-warmed fluid vs active fluid warming on the incidence of peri-operative hypothermia in short surgical procedures [Conference Paper]. Anaesthesia, 65(9), 942-945.

Ayhan, A., Balli, S. S., Camkiran Firat, A., & Kayhan, Z. (2018). Is hypothermia preventable during cesarean section under spinal anesthesia? [Article]. Anestezi Dergisi, 26(4), 238-244.

Azarmehr, T., Mofrad, Z. P., Mousavinasab, S. N., Nasiri, E., & Akbari, H. (2021). Effect of hypothermia prevention program on shivering and recovery time in patients undergoing abdominal surgery [Article]. Journal of Mazandaran University of Medical Sciences, 31(203), 180-186.

Bäcklund, M., Kellokumpu, I., Scheinin, T., von Smitten, K., Tikkanen, I., & Lindgren, L. (1998). Effect of temperature of insufflated CO2 during and after prolonged laparoscopic surgery [Article]. Surgical Endoscopy, 12(9), 1126-1130.

Baradaranfard, F., Ghadami, A., Jabalameli, M., & Aarabi, A. (2020). Comparing the efficacy of two warming methods on physiological indices of patients undergoing laparoscopic cholecystectomy [Article]. Koomesh, 22(1), 50-59.

Baradaranfard, F., Jabalameli, M., Ghadami, A., & Aarabi, A. (2019). Evaluation of Warming Effectiveness on Physiological Indices of Patients Undergoing Laparoscopic Cholecystectomy Surgery: A Randomized Controlled Clinical Trial [Randomized Controlled Trial]. Journal of PeriAnesthesia Nursing, 34(5), 1016-1024.

Bartosz, R., Agnieszka, R., Boleslaw, K., Francis, R., Przemyslaw, K., Andrzej, T., & Anna, J. (2016). A study of heat loss in patients undergoing general anesthesia warmed with a heated mattress with esophageal temperature monitoring compared to facial infrared thermography [Article]. Journal of Medical Imaging and Health Informatics, 6(1), 141-145.

Bayter-Marin, J. E., Cárdenas-Camarena, L., Durán, H., Valedon, A., Rubio, J., & Macias, A. A. (2018). Effects of Thermal Protection in Patients Undergoing Body Contouring Procedures: A Controlled Clinical Trial [Article]. Aesthetic Surgery Journal, 38(4), 448-456.

Becerra, Á., Cruz, R., Suárez, V., Betancor, V. D., Hussein, Z., & Rodríguez, A. (2013). Prevention of perioperative hypothermia in transurethral resection under spinal anesthesia [Conference Abstract]. European Journal of Anaesthesiology, 30, 19-20.

Beilin, B., Shavit, Y., Razumovsky, J., Wolloch, Y., Zeidel, A., & Bessler, H. (1998). Effects of mild perioperative hypothermia on cellular immune responses. Anesthesiology, 89(5), 1133-1140.

Benavides, R., Wong, A., & Nguyen, H. (2009). Improved outcomes for lap-banding using the insuflow® device compared with heated-only gas [Article]. Journal of the Society of Laparoendoscopic Surgeons, 13(3), 302-305.

Bennett, J., Ramachandra, V., Webster, J., & Carli, F. (1994). PREVENTION OF HYPOTHERMIA DURING HIP-SURGERY - EFFECT OF PASSIVE COMPARED WITH ACTIVE SKIN SURFACE WARMING. British Journal of Anaesthesia, 73(2), 180-183.

Benson, E. E., McMillan, D. E., & Ong, B. (2012). The effects of active warming on patient temperature and pain after total knee arthroplasty [Article]. The American journal of nursing, 112(5), 26-33; quiz 34, 42.

Bernard, J. M., Pinaud, M., & Souron, R. (1987). Peroperative hypothermia prevention [Clinical Trial; Journal Article; Randomized Controlled Trial]. Acta Anaesthesiologica Scandinavica, 31(6), 521‐523.

Berti, M., Casati, A., Torri, G., Aldegheri, G., Lugani, D., & Fanelli, G. (1997). Active warming, not passive heat retention, maintains normothermia during combined epidural-general anesthesia for hip and knee arthroplasty [Article]. Journal of Clinical Anesthesia, 9(6), 482-486.

Black, B., Chavda, M., English, S., & Sent-Doux, K. (2018). Effects of irrigation fluid temperature on post-operative temperature in transurethral resection of prostate: A randomised control trial [Conference Abstract]. BJU International, 121, 40.

Black, B., & English, S. (2019). Is turp safe with room temperature irrigation? : A randomised control trial [Conference Abstract]. Journal of Urology, 201(4), e643.

Board, T. N., & Srinivasan, M. S. (2008). The effect of irrigation fluid temperature on core body temperature in arthroscopic shoulder surgery [Article]. Archives of Orthopaedic and Trauma Surgery, 128(5), 531-533.

Boayam, W., Vichitvejpaisal, P., Suton, P., & Tapala, S. (2018). Comparison between forced air and intravenous fluid warmer in gynecologic laparoscopic surgery: A randomized trial [Article]. Journal of the Medical Association of Thailand, 101(8), 1005-1008.

Bock, M., Muller, J., Bach, A., Bohrer, H., Martin, E., & Motsch, J. (1998). Effects of preinduction and intraoperative warming during major laparotomy [Clinical Trial Randomized Controlled Trial]. British Journal of Anaesthesia, 80(2), 159-163.

Borbasi, S., & Brougham, L. (2002). Warming patients before clean surgery reduced the incidence of postoperative wound infection. Evidence Based Nursing, 48-48.

Borms, S. F., Engelen, S. L. E., Himpe, D. G. A., Suy, M. R. R., & Theunissen, W. J. H. (1994). Bair Hugger forced-air warming maintains normothermia more effectively than thermo-lite insulation [Article]. Journal of Clinical Anesthesia, 6(4), 303-307.

Brandes, I. F., Müller, C., Perl, T., Russo, S. G., Bauer, M., & Bräuer, A. (2013). Effektivität einer neuen Wärmedecke: Prospektive randomisierte Studie. Anaesthesist, 62(2), 137-142.

Brandt, S., Oguz, R., Hüttner, H., Waglechner, G., Chiari, A., Greif, R., Kurz, A., & Kimberger, O. (2010). Resistive-polymer versus forced-air warming: Comparable efficacy in orthopedic patients [Article]. Anesthesia and Analgesia, 110(3), 834-838.

Brodshaug, I., Tettum, B., & Raeder, J. (2019). Thermal Suit or Forced Air Warming in Prevention of Perioperative Hypothermia: A Randomized Controlled Trial [Randomized Controlled Trial]. Journal of PeriAnesthesia Nursing, 34(5), 1006-1015.

Brokelman, W. J. A., Holmdahl, L., Bergström, M., Falk, P., Klinkenbijl, J. H. G., & Reijnen, M. M. P. J. (2008). Heating of carbon dioxide during insufflation alters the peritoneal fibrinolytic response to laparoscopic surgery: A clinical trial [Conference Paper]. Surgical Endoscopy and Other Interventional Techniques, 22(5), 1232-1236.

Buraimoh, M. A., Nash, A., Howard, B., Yousaf, I., Koh, E., Banagan, K., Gelb, D., Schreibman, D., & Ludwig, S. C. (2019). Effect of forced-air warming blanket position in elective lumbar spine surgery: Intraoperative body temperature and postoperative complications [Article]. Surgical Neurology International, 10.

Butwick, A. J., Lipman, S. S., & Carvalho, B. (2007). Intraoperative forced air-warming during cesarean delivery under spinal anesthesia does not prevent maternal hypothermia [Comparative Study Randomized Controlled Trial Research Support, N.I.H., Extramural Research Support, Non-U.S. Gov't]. Anesthesia & Analgesia, 105(5), 1413-1419, table of contents.

Campos-Suarez, J. M., Casas-Vila, J. I., Litvan-Suquieni, H., & Villar-Landeira, J. M. (1997). [Air-convection heater for abdominal surgery. Study of the relation between surgical time and the efficacy of body temperature maintenance] [Clinical Trial Randomized Controlled Trial]. Revista Espanola de Anestesiologia y Reanimacion, 44(2), 47-51. (Calentador por conveccion de aire en cirugia abdominal)

Camus, Y., Delva, E., Bossard, A. E., Chandon, M., & Lienhart, A. (1997). Prevention of hypothermia by cutaneous warming with new electric blankets during abdominal surgery. BJA: The British Journal of Anaesthesia, 79(6), 796-797.

Camus, Y., Delva, E., Cohen, S., & Lienhart, A. (1996). The effects of warming intravenous fluids on intraoperative hypothermia and postoperative shivering during prolonged abdominal surgery [Clinical Trial Randomized Controlled Trial]. Acta Anaesthesiologica Scandinavica, 40(7), 779-782.

Camus, Y., Delva, E., Just, B., & Lienhart, A. (1993). Leg warming minimizes core hypothermia during abdominal surgery [Article]. Anesthesia and Analgesia, 77(5), 995-999.

Camus, Y., Delva, E., Sessler, D. I., & Lienhart, A. (1995). Pre-induction skin-surface warming minimizes intraoperative core hypothermia [Article]. Journal of Clinical Anesthesia, 7(5), 384-388.

Cantürk, M., Hakkı, M., Kocaoglu, N., Canturk, M., & Hakki, M. (2020). Effects of Isothermic Irrigation on Core Body Temperature During Endoscopic Urethral Stone Treatment Surgery Under Spinal Anesthesia: A Randomized Controlled Trial. Urology Journal, 17(1), 1-7.

Canturk, M., & Karbancioglu Canturk, F. (2019). Effects of isothermic crystalloid coload on maternal hypotension and fetal outcomes during spinal anesthesia for cesarean section: A randomized controlled trial [Article]. Taiwanese Journal of Obstetrics and Gynecology, 58(3), 428-433.

Carli, F., Clark, M. M., & Woollen, J. W. (1982). Investigation of the relationship between heat loss and nitrogen excretion in elderly patients undergoing major abdominal surgery under general anaesthetic. BJA: The British Journal of Anaesthesia, 54(10), 1023-1029.

Carli, F., Emery, P. W., & Freemantle, C. A. (1989). Effect of peroperative normothermia on postoperative protein metabolism in elderly patients undergoing hip arthroplasty. BJA: The British Journal of Anaesthesia, 63(3), 276-282.

Casati, A., Baroncini, S., Pattono, R., Fanelli, G., Bonarelli, S., Musto, P., Berti, M., & Torri, G. (1999). Effects of sympathetic blockade on the efficiency of forced-air warming during combined spinal-epidural anesthesia for total hip arthroplasty [Article]. Journal of Clinical Anesthesia, 11(5), 360-363.

Casati, A., Fanelli, G., Ricci, A., Musto, P., Cedrati, V., Altimari, G., Baroncini, S., Pattono, R., Montanini, S., & Torri, G. (1999). Shortening the discharging time after total hip replacement under combined spinal/epidural anesthesia by actively warming the patient during surgery [Article]. Minerva Anestesiologica, 65(7-8), 507-514.

Cavallini, M., Baruffaldi Preis, F. W., & Casati, A. (2005). Effects of mild hypothermia on blood coagulation in patients undergoing elective plastic surgery [Journal Article; Randomized Controlled Trial]. Plastic and Reconstructive Surgery, 116(1), 316‐321; discussion 322‐313.

Chakladar, A., Dixon, M. J., Crook, D., & Harper, C. M. (2012). Actively warming patients with a mattress during Caesarean section reduces the incidence of hypothermia and attenuates fall in haemoglobin [Conference Abstract]. Anaesthesia, 67, 14.

Chakladar, A., Dixon, M. J., Crook, D., & Harper, C. M. (2014). The effects of a resistive warming mattress during caesarean section: a randomised, controlled trial [Randomized Controlled Trial]. International Journal of Obstetric Anesthesia, 23(4), 309-316.

Chakladar, A., Dixon, M. J., & Harper, C. M. (2011). Warming mattress to prevent inadvertent perioperative hypothermia and shivering during elective Caesarean section [Conference Abstract]. British Journal of Anaesthesia, 107(2), 290P-291P.

Chataule, S. M., Meena, S. C., Jain, K., Hazarika, A., Luthra, A., Chauhan, R., & Sarna, R. (2021). Effect of preoperative forced air warming on perioperative hypothermia during femur fracture surgeries under neuraxial anaesthesia in elderly patients: a prospective randomized controlled trial [Conference Abstract]. Anesthesia and Analgesia, 133(3 SUPPL 2), 654.

Chebbout, R., Newton, R. S., Walters, M., Wrench, I. J., & Woolnough, M. (2017). Does the addition of active body warming to in-line intravenous fluid warming prevent maternal hypothermia during elective caesarean section? A randomised controlled trial [Randomized Controlled Trial]. International Journal of Obstetric Anesthesia, 31, 37-44.

Chen, S., Xu, W., Song, S., & Wang, X. (2020). Efficacy of self-heating calf sleeves for preventing deep vein thrombosis in lung cancer patients who undergo video-assisted thoracoscopic surgery lobectomy [Randomized Controlled Trial]. Annals of Palliative Medicine, 9(5), 2693-2698.

Chen, T. Y., Chen, K. T., Chang, D. P., Yeh, F. C., & Chang, C. L. (1994). The effect of heated humidifier in the prevention of intra-operative hypothermia [Clinical Trial Randomized Controlled Trial]. Acta Anaesthesiologica Sinica, 32(1), 27-30.

Chen, Z., Jiang, J., Dong, X., & Jiang, H. (2010). Effect of intraoperative warming on patient undergoing Le Fort I osteotomy [Article]. Shanghai kou qiang yi xue = Shanghai journal of stomatology, 19(2), 155-157.

Cheon, Y. M., & Yoon, H. (2017). [The Effects of 30-Minutes of Pre-Warming on Core Body Temperature, Systolic Blood Pressure, Heart Rate, Postoperative Shivering, and Inflammation Response in Elderly Patients with Total Hip Replacement under Spinal Anesthesia: A Randomized Double-blind Controlled Trial] [Randomized Controlled Trial]. Journal of Korean Academy of Nursing, 47(4), 456-466.

Cheong, J. Y., Chami, B., Fong, G. M., Wang, X. S., Keshava, A., Young, C. J., & Witting, P. (2020). Randomized clinical trial of the effect of intraoperative humidified carbon dioxide insufflation in open laparotomy for colorectal resection [Randomized Controlled Trial Research Support, Non-U.S. Gov't]. Bjs Open, 4(1), 45-58.

Cheong, J. Y., Keshava, A., & Young, C. (2019). The effect of humidified warmed CO2 during open colorectal surgery on body temperature: A randomized controlled trial [Conference Abstract]. Colorectal Disease, 21, 55.

ChiCTR-IOR-16008273. (2016a). Clinical study on the relationship between intraoperative warming and volume of blood loss and blood transfusion.

ChiCTR-IOR-16010198. (2016b). Study of warming methods in elderly patients.

ChiCTR-INR-17011621. (2017). Aggressive vs. standard body temperature management on myocardial injury in mid-aged and elderly patients receiving curative resection of esophageal carcinoma: a randomized controlled trial.

Choi, J. W., Kim, D. K., Lee, S. W., Park, J. B., & Lee, G. H. (2016). Efficacy of intravenous fluid warming during goal-directed fluid therapy in patients undergoing laparoscopic colorectal surgery: a randomized controlled trial [Randomized Controlled Trial]. Journal of International Medical Research, 44(3), 605-612.

Chung, S. H., Lee, B. S., Yang, H. J., Kweon, K. S., Kim, H. H., Song, J., & Shin, D. W. (2012). Effect of preoperative warming during cesarean section under spinal anesthesia [Journal: Article]. Korean Journal of Anesthesiology, 62(5), 454‐460.

Cobb, B., Cho, Y. R., Hilton, G., Ting, V., & Carvalho, B. (2016). Active Warming Utilizing Combined IV Fluid and Forced-Air Warming Decreases Hypothermia and Improves Maternal Comfort During Cesarean Delivery: A Randomized Control Trial. Anesthesia and Analgesia, 122(5), 1490-1497.

Cotoia, A., Mariotti, P. S., Ferialdi, C., Del Vecchio, P., Beck, R., Zaami, S., & Cinnella, G. (2021). Effectiveness of Combined Strategies for the Prevention of Hypothermia Measured by Noninvasive Zero-Heat Flux Thermometer During Cesarean Section [Article]. Frontiers in Medicine, 8.

Crivits, M., Reyntjens, K., Wouters, P., & De Hert, S. (2013). Comparison of two forced-air warming devices for the prevention of hypothermia during abdominal surgery in the Lloyd-Davies position [Conference Abstract]. European Journal of Anaesthesiology, 30, 21.

CTRI/2016/10/007418. (2016). A study to compare two techniques of warming the patient with warm air (starting warming 1 h before anesthesia and starting warming with start of anesthesia) to prevent reduction in patients body temperature during the surgery.

CTRI/2018/05/013886. (2018). Warming the patient before the surgery and look for fall in body temperature during the surgery and shivering after the surgery.

CTRI/2019/06/019576. (2019). To compare two types of warming blankets for prevention of hypothermia during colorectal surgery.

CTRI/2020/06/025755. (2020). Effect of pre-surgery warming on hypothermia during and after femur surgery under regional anaesthesia in old aged patients.

Davis, S. S., Mikami, D. J., Newlin, M., Needleman, B. J., Barrett, M. S., Fries, R., Larson, T., Dundon, J., Goldblatt, M. I., & Melvin, W. S. (2006). Heating and humidifying of carbon dioxide during pneumoperitoneum is not indicated: a prospective randomized trial [Randomized Controlled Trial Research Support, Non-U.S. Gov't]. Surgical Endoscopy, 20(1), 153-158.

de Bernardis, R. C. G., da Silva, M. P., Gozzani, J. L., Pagnocca, M. L., & Mathias, L. A. S. T. (2009). Use of forced-air to prevent intraoperative hypothermia [Article]. Revista Da Associacao Medica Brasileira, 55(4), 421-426.

de Bernardis, R. C. G., Siaulys, M. M., Vieira, J. E., & Mathias, L. (2016). Perioperative warming with a thermal gown prevents maternal temperature loss during elective cesarean section. A randomized clinical trial. Revista Brasileira de Anestesiologia, 66(5), 451-455.

De Mattia, A. L., Barbosa, M. H., de Freitas, J. P. A., Rocha, A. D., & Pereira, N. H. C. (2013). Warmed intravenous infusion for controlling intraoperative hypothermia. Revista Latino-Americana de Enfermagem, 21(3), 803-810.

de Oliveira, S. A. R., Lucio, L. M. C., Modolo, N. S. P., Hayashi, Y., Braz, M. G., de Carvalho, L. R., Braz, L. G., & Braz, J. R. C. (2017). The Humidity in a Low-Flow Drager Fabius Anesthesia Workstation with or without Thermal Insulation or a Heat and Moisture Exchanger: A Prospective Randomized Clinical Trial. PLoS ONE, 12(1).

De Witte, J. L., Demeyer, C., & Vandemaele, E. (2010). Resistive-heating or forced-air warming for the prevention of redistribution hypothermia [Article]. Anesthesia and Analgesia, 110(3), 829-833.

Degirmenci, A. K., Ozkardesler, S., Terzi, C., Arslan, N. C., Ocmen, E., Atlia, K., Unek, T., & Canda, A. E. (2015). Effect of standard normothermia protocol on surgical site infections: Preliminary results of a randomized controlled trial [Conference Abstract]. European Surgery - Acta Chirurgica Austriaca, 47, S262.

Deriaz, H., Fiez, N., & Lienhart, A. (1992). Comparative effects of a hygrophobic filter and a heated humidifier on intraoperative hypothermia [Article]. Annales Francaises d'Anesthesie et de Reanimation, 11(2), 145-149.

Dostálová, V., Schreiberova, J., Bartoš, M., Česák, T., Habalová, J., Dostálová, V., & Dostál, P. (2017). Thermal management in patients undergoing elective spinal surgery in prone position - A prospective randomized trial [Article]. Ceska a Slovenska Neurologie a Neurochirurgie, 80(5), 553-560.

Drks. (2012). Effectivness of a new conductive prewarming system to prevent perioperativ hypothermia in ENT surgery.

Drks. (2013). Convective vs. conductive patient warming - comparison of efficacy for preventing perioperative hypothermia in patients undergoing mininmal invasive thoracic surgery.

Egan, C., Bernstein, E., Reddy, D., Ali, M., Paul, J., Yang, D. S., & Sessler, D. I. (2011). A Randomized Comparison of Intraoperative PerfecTemp and Forced-Air Warming During Open Abdominal Surgery. Anesthesia and Analgesia, 113(5), 1076-1081.

Emmert, A., Franke, R., Brandes, I. F., Hinterthaner, M., Danner, B. C., Bauer, M., & Brauer, A. (2017). Comparison of Conductive and Convective Warming in Patients Undergoing Video-Assisted Thoracic Surgery: A Prospective Randomized Clinical Trial [Comparative Study Randomized Controlled Trial]. Thoracic & Cardiovascular Surgeon, 65(5), 362-366.

Erdling, A., & Johansson, A. (2015). Core Temperature—The Intraoperative Difference Between Esophageal Versus Nasopharyngeal Temperatures and the Impact of Prewarming, Age, and Weight: A Randomized Clinical Trial. AANA Journal, 83(2), 99-105.

Eun-Hui, S., & Mi-Young, C. (2019). Effects of Warmed Fluid Irrigation Intervention and Forced-Air Warming Intervention on Hypothermia in Transurethral Operation Under Spinal Anesthesia. Medico-Legal Update, 19(1), 618-622.

Fabregas, N., Salazar, F., & Do, M. (2009). Intraoperative warming is an independent risk factor of postoperative cognitive dysfunction [Conference Abstract]. Journal of Neurosurgical Anesthesiology, 21(4), 408.

Fallis, W. M., Hamelin, K., Symonds, J., & Wang, X. (2006). Maternal and newborn outcomes related to maternal warming during cesarean delivery [Journal Article; Randomized Controlled Trial; Research Support, Non‐U.S. Gov't]. Journal of obstetric, gynecologic, and neonatal nursing : JOGNN, 35(3), 324‐331.

Fallon, J. (2021). Pre-operative arm warming improving long term fistula patency [Conference Abstract]. Journal of Vascular Access, 22(6), 15NP.

Fanelli, A., Danelli, G., Ghisi, D., Ortu, A., Moschini, E., & Fanelli, G. (2009). The efficacy of a resistive heating under-patient blanket versus a forced-air warming system: a randomized controlled trial [Comparative Study Randomized Controlled Trial Research Support, Non-U.S. Gov't]. Anesthesia & Analgesia, 108(1), 199-201.

Farley, D. R., Greenlee, S. M., Larson, D. R., & Harrington, J. R. (2004). Double-blind, prospective, randomized study of warmed, humidified carbon dioxide insufflation vs standard carbon dioxide for patients undergoing laparoscopic cholecystectomy. Archives of Surgery, 139(7), 739-744.

Fettes, S., Mulvaine, M., & Van Doren, E. (2013). Effect of preoperative forced-air warming on postoperative temperature and postanesthesia care unit length of stay [Randomized Controlled Trial]. AORN Journal, 97(3), 323-328.

Fleisher, L. A., Metzger, S. E., Lam, J., & Harris, A. (1998). Perioperative cost-finding analysis of the routine use of intraoperative forced-air warming during general anesthesia [Clinical Trial Comparative Study Randomized Controlled Trial]. Anesthesiology, 88(5), 1357-1364.

Fossum, S., Hays, J., & Henson, M. M. (2001). A comparison study on the effects on prewarming patients in the outpatient surgery setting. Journal of PeriAnesthesia Nursing, 16(3), 187-194.

Frank, S. M., Beattie, C., Christopherson, R., Norris, E. J., Perler, B. A., Williams, G. M., & Gottlieb, S. O. (1993). Unintentional hypothermia is associated with postoperative myocardial ischemia. The Perioperative Ischemia Randomized Anesthesia Trial Study Group [Clinical Trial Randomized Controlled Trial Research Support, U.S. Gov't, P.H.S.]. Anesthesiology, 78(3), 468-476.

Frank, S. M., Higgins, M. S., Breslow, M. J., Fleisher, L. A., Gorman, R. B., Sitzmann, J. V., Raff, H., & Beattie, C. (1995). The catecholamine, cortisol, and hemodynamic responses to mild perioperative hypothermia. A randomized clinical trial [Clinical Trial Comparative Study Randomized Controlled Trial Research Support, Non-U.S. Gov't Research Support, U.S. Gov't, P.H.S.]. Anesthesiology, 82(1), 83-93.

Franke, R., Bräuer, A., Emmert, A., Hinterthaner, M., Brandes, I., Quintel, M., Danner, B., & Schöndube, F. (2015). Prevention of perioperative hypothermia in vats: A prospective randomised controlled trial comparing forced-air warming with conductive warming [Conference Abstract]. Thoracic and Cardiovascular Surgeon, 63.

Frey, J. M., Janson, M., Svanfeldt, M., Svenarud, P. K., & van der Linden, J. A. (2012a). Intraoperative local insufflation of warmed humidified CO₂ increases open wound and core temperatures: a randomized clinical trial [Journal Article; Randomized Controlled Trial; Research Support, Non‐U.S. Gov't]. World Journal of Surgery, 36(11), 2567‐2575.

Frey, J. M., Janson, M., Svanfeldt, M., Svenarud, P. K., & van der Linden, J. A. (2012b). Local insufflation of warm humidified CO₂increases open wound and core temperature during open colon surgery: a randomized clinical trial [Journal Article; Randomized Controlled Trial; Research Support, Non‐U.S. Gov't]. Anesthesia and Analgesia, 115(5), 1204‐1211.

Gherghina, V., Cindea, I., & Balcan, A. (2019). Does warmed intravenous fluid reduces shivering after general anesthesia in elderly? [Conference Abstract]. Intensive Care Medicine Experimental, 7.

Goldberg, M. E., Epstein, R., Rosenblum, F., Larijani, G. E., Marr, A., Lessin, J., Torjman, M., & Seltzer, J. (1992). Do heated humidifiers and heat and moisture exchangers prevent temperature drop during lower abdominal surgery? [Clinical Trial Comparative Study Randomized Controlled Trial]. Journal of Clinical Anesthesia, 4(1), 16-20.

Gozubuyuk, E., Aygun, E., Basaran, I., Canbolat, N., Cavdaroglu, B., Akgul, T., & Buget, M. I. (2021). Effects of Changes in Body Temperature on Perioperative Bleeding in Adolescent Idiopathic Scoliosis Surgery [Article in Press]. Therapeutic Hypothermia and Temperature Management.

Grant, E. N., Craig, M. G., Tao, W., McIntire, D. D., & Leveno, K. J. (2015). Active Warming during Cesarean Delivery: Should We SCIP It? [Article]. American Journal of Perinatology, 32(10), 933-938.

Gulia, A., Gupta, N., Kumar, V., Bhoriwal, S., Malhotra, R. K., Bharti, S. J., Garg, R., Mishra, S., & Bhatnagar, S. (2021). Comparison of two forced air warming systems for prevention of intraoperative hypothermia in carcinoma colon patients: a prospective randomized study [Journal: Article in Press]. Journal of Clinical Monitoring and Computing.

Gulucu, S., & Cakmak, B. (2021). Warm distension fluid reduces pain severity in office hysteroscopy: a randomized controlled trial. ANNALS OF SAUDI MEDICINE, 41(3), 135-140.

Guo, Z. X., & Zheng, H. (2019). [Effect of intraoperative warming on muscle relaxation recovery of cisatracurium in patients undergoing gastrointestinal surgery] [Randomized Controlled Trial]. Chung-Hua i Hsueh Tsa Chih [Chinese Medical Journal], 99(35), 2777-2780.

Gupta, N., Bharti, S., Kumar, V., Garg, R., Mishra, S., & Bhatnagar, S. (2019). Comparative evaluation of forced air warming and infusion of amino acid-enriched solution on intraoperative hypothermia in patients undergoing head and neck cancer surgeries: A prospective randomised study [Article]. Saudi Journal of Anaesthesia, 13(4), 318-324.

Hamada, Y., Ouchi, T., Kato, T., Agata, H., Serita, R., & Koitabashi, T. (2010). Upper type forced-air warming blanket with the temperature setting of 38degreeC might be a better choice for maintaining normothermia [Journal: Conference Abstract]. Anesthesia and Analgesia, 110(3 SUPPL. 1), S246.

Hamza, M. A., Schneider, B. E., White, P. F., Recart, A., Villegas, L., Ogunnaike, B., Provost, D., & Jones, D. (2005). Heated and humidified insufflation during laparoscopic gastric bypass surgery: effect on temperature, postoperative pain, and recovery outcomes [Clinical Trial Randomized Controlled Trial Research Support, Non-U.S. Gov't]. Journal of Laparoendoscopic & Advanced Surgical Techniques. Part A, 15(1), 6-12.

Han, S. B., Gwak, M. S., Choi, S. J., Kim, M. H., Ko, J. S., Kim, G. S., & Joo, H. S. (2013). Effect of active airway warming on body core temperature during adult liver transplantation [Randomized Controlled Trial]. Transplantation Proceedings, 45(1), 251-254.

Hara, K., Kuroda, H., Matsuura, E., Ishimatsu, Y., Honda, S., Takeshita, H., & Sawai, T. (2022). Underbody blankets have a higher heating effect than overbody blankets in lithotomy position endoscopic surgery under general anesthesia: a randomized trial [Randomized Controlled Trial]. Surgical Endoscopy, 36(1), 670-678.

Hasankhani, H., Mohammadi, E., Moazzami, F., Mokhtari, M., & Naghgizadh, M. M. (2007). The effects of intravenous fluids temperature on perioperative hemodynamic situation, post-operative shivering, and recovery in orthopaedic surgery [Randomized Controlled Trial]. Canadian Operating Room Nursing Journal, 25(1), 20-24, 26-27.

Hasegawa, K., Nakagawa, F., Negishi, C., & Ozaki, M. (2012). Core temperatures during major abdominal surgery in patients warmed with new circulating-water garment, forced-air warming, or carbon-fiber resistive-heating system [Article]. Journal of Anesthesia, 26(2), 168-173.

Hasegawa, K., Negishi, C., Nakagawa, F., Mukai, S., & Ozaki, M. (2003). [The efficacy of carbon-fiber resistive-heating in prevention of core hypothermia during major abdominal surgery] [Clinical Trial Randomized Controlled Trial]. Masui - Japanese Journal of Anesthesiology, 52(6), 636-641.

Hassani, V., Chaichian, S., Rahimizadeh, A., Darabi, M. E., Nobahar, M. R., Moghaddam, M. J., Homaie, M., Fotouhi, G., Alimohamadi, Y., & Moradi, Y. (2018). Comparative study of the effect of warming at various temperatures on biochemical, hematologic, and hemodynamic parameters during spinal fusion surgery under intravenous anesthesia [Article]. Anesthesiology and Pain Medicine, 8(4).

He, Y., Feng, Y. G., He, J., Liang, B., Jiang, M. D., Liu, J., Kang, Y. M., Ma, L. P., Zhang, Q., Peng, Q. J., & et al. (2021). Effects of irrigation fluid temperature during flexible ureteroscopic holmium laser lithotripsy on postoperative fever and shivering: a randomized controlled trial [Journal Article; Randomized Controlled Trial]. BMC Urology, 21(1), 72.

Hong-xia, X., Zhi-jian, Y., Hong, Z., & Zhiqing, L. (2010). Prevention of hypothermia by infusion of warm fluid during abdominal surgery. Journal of PeriAnesthesia Nursing, 25(6), 366-370.

Horn, E.-P., Bein, B., Broch, O., Iden, T., Böhm, R., Latz, S.-K., & Höcker, J. (2016). Warming before and after epidural block before general anaesthesia for major abdominal surgery prevents perioperative hypothermia: A randomised controlled trial. European Journal of Anaesthesiology (Cambridge University Press), 33(5), 334-340.

Horn, E. P., Bein, B., Bohm, R., Steinfath, M., Sahili, N., & Hocker, J. (2012). The effect of short time periods of pre-operative warming in the prevention of peri-operative hypothermia [Randomized Controlled Trial]. Anaesthesia, 67(6), 612-617.

Horn, E. P., Schroeder, F., Gottschalk, A., Sessler, D. I., Hiltmeyer, N., Standl, T., & Schulte, J. (2002). Active warming during cesarean delivery. Anesthesia and Analgesia, 94(2), 409-414.

Hosseini, S. R., Mohseni, M. G., Aghamir, S. M. K., & Rezaei, H. (2019). Effect of Irrigation Solution Temperature on Complications of Percutaneous Nephrolithotomy: A Randomized Clinical Trial. Urology Journal, 16(6), 525-529.

Hovmann Rasmussen, Y., Leikersfeldt, G., & Drenck, N. E. (1998). Forced-air surface warming versus oesophageal heat exchanger in the prevention of peroperative hypothermia [Article]. Acta Anaesthesiologica Scandinavica, 42(3), 348-352.

Hynson, J. M., & Sessler, D. I. (1992). Intraoperative warming therapies: A comparison of three devices [Article]. Journal of Clinical Anesthesia, 4(3), 194-199.

Ihn, C. H., Joo, J. D., Chung, H. S., Choi, J. W., Kim, D. W., Jeon, Y. S., Kim, Y. S., & Choi, W. Y. (2008). Comparison of three warming devices for the prevention of core hypothermia and post-anaesthesia shivering [Article]. Journal of International Medical Research, 36(5), 923-931.

Irct201601163311N. (2016). Effect of intravenous warm fluids on surgery shivering and core temperature in patients undergoing abdominal surgery.

Irct2013092114722N. (2013). Comparison of the efficacy of two methods of warming on patient in abdominal surgery on physiologic parameters and shivering in hamedan´s besat hospital in 1392.

Irct2016042527597N. (2016). Prevention of Postoprative Shivering.

Irct2017041533435N. (2017). Comparison the effect of two methods of warming on the physiological indices during and after laparoscopic abdominal surgery at the Alzahra Hospital of Isfahan in 2017.

Işıklı, A. G., & Fındık, Ü. (2022). Determining the Effectiveness of Forced-Air Warming Blankets in Maintaining Postoperative Body Temperature: A Randomized Controlled Trial [Article in Press]. Journal of perianesthesia nursing : official journal of the American Society of PeriAnesthesia Nurses.

Isrctn. (2001). The effect of pre-operative warming on wound infection rates after clean surgery.

Isrctn. (2003). The effect of systematic warming (Pegasus-Inditherm mattress) on outcomes of elective major surgery.

Isrctn. (2006). A comparison of the ability of the Inditherm® mattress and the forced hot air blower to prevent hypothermia.

Iwasaka, H., Ishida, K., Kitano, T., Mizutani, A., Taniguchi, K., & Honda, N. (1992). [Heat conservation during abdominal surgery] [Clinical Trial Comparative Study Randomized Controlled Trial]. Masui - Japanese Journal of Anesthesiology, 41(4), 666-669.

Jae Hwa Yoo, S. Y. O., Sang Ho Kim, Sun Young Park, Yoo-mi Han, Doyeon Kim. (2018). The effect of 10 minutes of prewarming for prevention of inadvertent perioperative hypothermia: comparison with 30 minutes of prewarming [Original Article]. Anesthesiology and Pain Medicine, 13(4), 447‐453.

Jaffe, J. S., McCullough, T. C., Harkaway, R. C., & Ginsberg, P. C. (2001). Effects of irrigation fluid temperature on core body temperature during transurethral resection of the prostate [Article]. Urology, 57(6), 1078-1081.

Janicki, P. K., Higgins, M. S., Janssen, J., Johnson, R. F., & Beattie, C. (2001). Comparison of two different temperature maintenance strategies during open abdominal surgery: Upper body forced-air warming versus whole body water garment [Article]. Anesthesiology, 95(4), 868-874.

Janicki, P. K., Stoica, C., Chapman, W. C., Wright, J. K., Walker, G., Pai, R., Walia, A., Pretorius, M., & Pinson, C. W. (2002). Water warming garment versus forced air warming system in prevention of intraoperative hypothermia during liver transplantation: A randomized controlled trial [ISRCTN32154832] [Article]. BMC Anesthesiology, 2.

Jiang, R., Sun, Y., Wang, H., Liang, M., Xie, X., & Ting, C. K. (2019). Effect of different carbon dioxide (CO2) insufflation for laparoscopic colorectal surgery in elderly patients: A randomized controlled trial [Article]. Medicine (United States), 98(41).

Jo, Y. Y., Chang, Y. J., Kim, Y. B., Lee, S., & Kwak, H. J. (2015). Effect of Preoperative Forced-Air Warming on Hypothermia in Elderly Patients Undergoing Transurethral Resection of the Prostate [Randomized Controlled Trial]. Urology Journal, 12(5), 2366-2370.

Jo, Y. Y., Kim, H. S., Chang, Y. J., Yun, S. Y., & Kwak, H. J. (2013). The effect of warmed inspired gases on body temperature during arthroscopic shoulder surgery under general anesthesia [Article]. Korean Journal of Anesthesiology, 65(1), 14-18.

Johansson, T., Lisander, B., & Ivarsson, I. (1999). Mild hypothermia does not increase blood loss during total hip arthroplasty [Article]. Acta Anaesthesiologica Scandinavica, 43(10), 1005-1010.

John, J., Kader, F., & Ockendon, M. (2019). Evaluation of an alternative to forced air warming for temperature management in major spine surgery [Conference Abstract]. Anaesthesia, 74, 24.

John, M., Crook, D., Dasari, K., Eljelani, F., El-Haboby, A., & Harper, C. M. (2016). Comparison of resistive heating and forced-air warming to prevent inadvertent perioperative hypothermia [Comparative Study Randomized Controlled Trial]. British Journal of Anaesthesia, 116(2), 249-254.

Joo, E., Seo, H., Kim, Y. K., & Hwang, J. H. (2014). The effect of electrically heated humidifier to intraoperative temperature management in elderly patients receiving open abdominal surgery [Conference Abstract]. European Journal of Anaesthesiology, 31, 29-30.

Joo, Y., Kim, H. J., Kim, J. T., Kim, H. S., Lee, S. C., & Kim, C. S. (2009). Effect of active warming on shivering during spinal anesthesia. Korean Journal of Anesthesiology, 57(2), 176‐180.

JPRN-UMIN000015776. (2014). Effect of warming on genral anesthetics requirement for loss of consciousness.

JPRN-UMIN000016881. (2015a). Prewarming prevents perioperative hypothermia.

JPRN-UMIN000019858. (2015b). Thermal effect of air warmer blanket on donor of living liver transplantation.

Jun, J.-H., Chung, M. H., Kim, E. M., Jun, I.-J., Kim, J. H., Hyeon, J.-S., Lee, M. H., Lee, H. S., & Choi, E. M. (2018). Effect of pre-warming on perioperative hypothermia during holmium laser enucleation of the prostate under spinal anesthesia: a prospective randomized controlled trial. BMC Anesthesiology, 18(1), N.PAG-N.PAG.

Jun, J. H., Chung, M. H., Jun, I. J., Kim, Y., Kim, H., Kim, J. H., Choi, Y. R., & Choi, E. M. (2019). Efficacy of forced-air warming and warmed intravenous fluid for prevention of hypothermia and shivering during caesarean delivery under spinal anaesthesia: A randomised controlled trial [Randomized Controlled Trial Research Support, Non-U.S. Gov't]. European Journal of Anaesthesiology, 36(6), 442-448.

Jung, K. T., Kim, S. H., & So, K. Y. (2014). Laboratory and clinical effect of newly degined fluid warming kit using the humidified and heated circuit [Conference Abstract]. European Journal of Anaesthesiology, 31, 36.

Jung, K. T., Kim, S. H., So, K. Y., So, H. J., & Shim, S. B. (2015). Clinical evaluation of a newly designed fluid warming kit on fluid warming and hypothermia during spinal surgery [Article]. Korean Journal of Anesthesiology, 68(5), 462-468. h

Just, B., Trevien, V., Delva, E., & Lienhart, A. (1993). Prevention of intraoperative hypothermia by preoperative skin-surface warming [Clinical Trial Randomized Controlled Trial]. Anesthesiology, 79(2), 214-218.

Kabbara, A., Goldlust, S. A., Smith, C. E., Hagen, J. F., & Pinchak, A. C. (2002). Randomized prospective comparison of forced air warming using hospital blankets versus commercial blankets in surgical patients [Article]. Anesthesiology, 97(2), 338-344.

Kadam, V. R., Moyes, D., & Moran, J. L. (2009). Relative efficiency of two warming devices during laparoscopic cholecystectomy [Comparative Study; Journal Article; Randomized Controlled Trial]. Anaesthesia and Intensive Care, 37(3), 464‐468.

Kalev, G., Egglseder, T., Marquardt, C., & Schiedeck, T. (2020). Influence of Local Insufflation of Warm Humidified CO2 on the Wound Surface and Body Core Temperature as well as on Wound Healing in Open Colorectal Surgery. Zentralblatt fur Chirurgie, 145(2), 188-199.

Kamitani, K., Higuchi, A., Takebayashi, T., Miyamoto, Y., & Yoshida, H. (1999). Covering the head and face maintains intraoperative core temperature [Article]. Canadian Journal of Anaesthesia, 46(7), 649-652.

Kang, S. K., & Park, S. (2020). Effect of the ASPAN Guideline on Perioperative Hypothermia Among Patients With Upper Extremity Surgery Under General Anesthesia: A Randomized Controlled Trial. Journal of PeriAnesthesia Nursing, 35(3), 298-306.

Karayan, J., Thomas, D., Lacoste, L., Dhoste, K., Ricco, J. B., & Fusciardi, J. (1996). Delayed forced air warming prevents hypothermia during abdominal aortic surgery [Clinical Trial Randomized Controlled Trial]. British Journal of Anaesthesia, 76(3), 459-460.

Kati, B., Buyukfirat, E., Pelit, E. S., Yagmur, I., Demir, M., Albayrak, I. H., & Ciftci, H. (2018). Percutaneous Nephrolithotomy with Different Temperature Irrigation and Effects on Surgical Complications and Anesthesiology Applications [Article]. Journal of Endourology, 32(11), 1050-1053.

Kaudasch, G., Schempp, P., Skierski, P., & Turner, E. (1996). Effect of forced-air warming during abdominal surgery on the early postoperative heat balance of ventilated patients [Article]. Anaesthesist, 45(11), 1075-1081.

Kaufner, L., Niggemann, P., Baum, T., Casu, S., Sehouli, J., Bietenbeck, A., Boschmann, M., Spies, C. D., Henkelmann, A., & von Heymann, C. (2019). Impact of brief prewarming on anesthesia-related core-temperature drop, hemodynamics, microperfusion and postoperative ventilation in cytoreductive surgery of ovarian cancer: a randomized trial [Randomized Controlled Trial Research Support, Non-U.S. Gov't]. BMC Anesthesiology, 19(1), 161.

Kct. (2018). Effect of prewarming using a forced air blanket on body core temperature during adult liver transplantation. https://trialsearch.who.int/Trial2.aspx?TrialID=KCT0003230.

Kelly, J. A., Doughty, J. K., Hasselbeck, A. N., & Vacchiano, C. A. (2000). The effect of arthroscopic irrigation fluid warming on body temperature [Clinical Trial Randomized Controlled Trial]. Journal of PeriAnesthesia Nursing, 15(4), 245-252.

Kim, D. K., Rhee, K. Y., Kwon, W. K., Kim, T. Y., & Kang, J. E. (2007). A heated humidifier does not reduce laryngo- pharyngeal complaints after brief laryngeal mask anesthesia [Randomized Controlled Trial Research Support, Non-U.S. Gov't]. Canadian Journal of Anaesthesia, 54(2), 134-140.

Kim, E., Lee, S. Y., Lim, Y. J., Choi, J. Y., Jeon, Y. T., Hwang, J. W., & Park, H. P. (2015). Effect of a new heated and humidified breathing circuit with a fluid-warming device on intraoperative core temperature: a prospective randomized study [Randomized Controlled Trial Research Support, Non-U.S. Gov't]. Journal of Anesthesia, 29(4), 499-507.

Kim, G., Kim, M. H., Lee, S. M., Choi, S. J., Shin, Y. H., & Jeong, H. J. (2014). Effect of pre-warmed intravenous fluids on perioperative hypothermia and shivering after ambulatory surgery under monitored anesthesia care [Randomized Controlled Trial]. Journal of Anesthesia, 28(6), 880-885.

Kim, H. J., Jeon, G. E., Choi, J. M., Jeong, S. M., Seong, K. W., & Yang, H. S. (2008). The Effects of Temperature Monitoring Methods and Thermal Management Methods during Spinal Surgery. Korean Journal of Anesthesiology, 54(6), 623‐628.

Kim, H. J., Oh, J. E., Koh, W. U., Ro, Y. J., & Yang, H. S. (2016). Comparison of warming methods for core temperature preservation during total knee arthroplasty using a pneumatic tourniquet. Anesthesiology and Pain Medicine, 11(1), 91‐98.

Kim, M. H., & Kang, Y. L. (2002). Effects of Intraoperative Hypothermia on the Incidence of Post-operative Infection and Responses of Peripheral White Blood Cells. Korean Journal of Anesthesiology, 43(6), 742‐748.

Kim, S., Baek, W. Y., Jeon, Y. H., Lim, D. G., & Park, S. S. (2012). Comparisons of three different warming devices on body temperature changes during open gastrectomy [Conference Abstract]. British Journal of Anaesthesia, 108, ii133-ii134.

Kim Unjin, L. Y. M. (2017). The Effects of Active Warming on Pain, Temperature, and Thermal Discomfort in Postoperative Patients after General Anesthesia for Abdominal Surgery [Original Article]. J korean crit care nurs, 10(3), 53‐64.

Kim, Y. S., Jeon, Y. S., Lee, J. A., Park, W. K., Koh, H. S., Joo, J. D., In, J. H., & Seo, K. W. (2009). Intra-operative warming with a forced-air warmer in preventing hypothermia after tourniquet deflation in elderly patients [Article]. The Journal of international medical research, 37(5), 1457-1464.

Kim, Y. S., Lee, J. Y., Yang, S. C., Song, J. H., Koh, H. S., & Park, W. K. (2009). Comparative Study of the Influence of Room-Temperature and Warmed Fluid Irrigation on Body Temperature in Arthroscopic Shoulder Surgery [Article]. Arthroscopy - Journal of Arthroscopic and Related Surgery, 25(1), 24-29.

Kimberger, O., Illievich, U., & Lenhardt, R. (2007). The effect of skin surface warming on pre-operative anxiety in neurosurgery patients [Randomized Controlled Trial]. Anaesthesia, 62(2), 140-145.

Kissler, S., Haas, M., Strohmeier, R., Schmitt, H., Rody, A., Kaufmann, M., & Siebzehnruebl, E. (2004). Effect of humidified and heated CO2 during gynecologic laparoscopic surgery on analgesic requirements and postoperative pain [Article]. Journal of the American Association of Gynecologic Laparoscopists, 11(4), 473-477.

Klugsberger, B., Schreiner, M., Rothe, A., Haas, D., Oppelt, P., & Shamiyeh, A. (2014). Warmed, humidified carbon dioxide insufflation versus standard carbon dioxide in laparoscopic cholecystectomy: a double-blinded randomized controlled trial. Surgical Endoscopy, 28(9), 2656-2660.

Koc, B. B., Schotanus, M. G. M., Kollenburg, J.-P. A. P. A. C., Janssen, M. J. A., Tijssen, F., & Jansen, E. J. P. (2017). Effectiveness of Early Warming With Self-Warming Blankets on Postoperative Hypothermia in Total Hip and Knee Arthroplasty. Orthopaedic Nursing, 36(5), 356-360.

Konrad, F., Mezödy, M., Goertz, A., Marx, T., & Georgieff, M. (1996). The effect of a heat and moisture exchanger (HME) on bronchial mucus transport in a closed inhalation anesthesia system [Clinical Trial; English Abstract; Journal Article; Randomized Controlled Trial]. Der Anaesthesist, 45(9), 802‐806.

Krenzischek, D. A., Frank, S. M., & Kelly, S. (1995). Forced-air warming versus routine thermal care and core temperature measurement sites [Article]. Journal of Post Anesthesia Nursing, 10(2), 69-78.

Kristensen, G., Guldager, H., & Gravesen, H. (1986). Prevention of peroperative hypothermia in abdominal surgery [Clinical Trial Randomized Controlled Trial Research Support, Non-U.S. Gov't]. Acta Anaesthesiologica Scandinavica, 30(4), 314-316.

Kulkarni, P., Webster, J., & Carli, F. (1995). Body heat transfer during hip surgery using active core warming [Article]. Canadian Journal of Anaesthesia, 42(7), 571-576.

Kumar, S., Wong, P., Bohra, D. J., & Leaper, D. J. (2004). Effect of peri-operative systemic warming on outcomes after elective major abdominla surgery. A randomised controlled trial. 2nd world union of wound healing societies meeting; 2004 ,8-13 july; paris, 6, Abstract No. A003.

Kümin, M., Deery, J., Turney, S., Price, C., Vinayakam, P., Smith, A., Filippa, A., Wilkinson-Guy, L., Moore, F., O'Sullivan, M., & et al. (2019). Reducing Implant Infection in Orthopaedics (RIIiO): results of a pilot study comparing the influence of forced air and resistive fabric warming technologies on postoperative infections following orthopaedic implant surgery [Comparative Study; Journal Article; Randomized Controlled Trial]. Journal of Hospital Infection, 103(4), 412‐419.

Kurz, A., Kurz, M., Poeschl, G., Faryniak, B., Redl, G., & Hackl, W. (1993). Forced-air warming maintains intraoperative normothermia better than circulating-water mattresses [Clinical Trial Comparative Study Randomized Controlled Trial Research Support, Non-U.S. Gov't]. Anesthesia & Analgesia, 77(1), 89-95.

Kurz, A., Sessler, D. I., Narzt, E., Bekar, A., Lenhardt, R., Heumer, G., & Lackner, F. (1995). POSTOPERATIVE HEMODYNAMIC AND THERMOREGULATORY CONSEQUENCES OF INTRAOPERATIVE CORE HYPOTHERMIA. Journal of Clinical Anesthesia, 7(5), 359-366.

Kwang-seob Shin, G. Y. L., Eun Hee Chun, Youn Jin Kim, Won Joong Kim. (2017). Effect of short-term prewarming on body temperature in arthroscopic shoulder surgery [Original Article]. Anesthesiology and Pain Medicine, 12(4), 388‐393.

Large, T., Nottingham, C. U., & Krambeck, A. (2019). Initial experience with intraoperative fluid warming during holmium laser enucleation of the prostate (HoLEP) [Conference Abstract]. Journal of Endourology, 33, A111.

Lau, A., Lowlaavar, N., Cooke, E. M., West, N., German, A., Morse, D. J., Görges, M., & Merchant, R. N. (2018). Effet du réchauffement préopératoire sur l’hypothermie peropératoire: essai randomisé contrôlé. Canadian Journal of Anaesthesia, 65(9), 1029-1040.

Lauronen, S. L., Makinen, M. T., Annila, P., Huhtala, H., Yli-Hankala, A., & Kalliomaki, M. L. (2021). Thermal suit connected to a forced-air warming unit for preventing intraoperative hypothermia: A randomised controlled trial [Randomized Controlled Trial]. Acta Anaesthesiologica Scandinavica, 65(2), 176-181.

Leben, J., & Tryba, M. (1997). Prevention of hypothermia during surgery. Contribution of convective heating system and warm infusion [Clinical Trial; Journal Article; Randomized Controlled Trial]. Annals of the New York Academy of Sciences, 813, 807‐811.

Lee, H. J., Kim, K. S., Jeong, J. S., Kim, K. N., & Lee, B. C. (2015). The influence of mild hypothermia on reversal of rocuronium-induced deep neuromuscular block with sugammadex [Article]. BMC Anesthesiology, 15(1).

Lee, H. K., Jang, Y. H., Choi, K. W., & Lee, J. H. (2011). The effect of electrically heated humidifier on the body temperature and blood loss in spinal surgery under general anesthesia [Article]. Korean Journal of Anesthesiology, 61(2), 112-116.

Lee, J. H., Kim, H. J., Seo, H. J., Choi, Y. J., Ro, Y. J., & Yang, H. S. (2013). The effects of the warming devices in patients undergoing tourniquet technique for total knee arthroplasty under the general anesthesia [Conference Abstract]. European Journal of Anaesthesiology, 30, 18-19.

Lee, K. C., Kim, J. Y., Kwak, H. J., Lee, H. D., & Kwon, I. W. (2011). The effect of heating insufflation gas on acid-base alterations and core temperature during laparoscopic major abdominal surgery [Article]. Korean Journal of Anesthesiology, 61(4), 275-280.

Lee, L., Leslie, K., Kayak, E., & Myles, P. S. (2004). Intraoperative patient warming using radiant warming or forced-air warming during long operations [Article]. Anaesthesia and Intensive Care, 32(3), 358-361.

Lee Min Ji, J. J. H. (2020). Effect of Preoperative Warming on Prevention of Hypothermia during Surgery in Patients with Total Hip Replacement Arthroplasty under Spinal Anesthesia [Original Article]. J korean clin nurs res, 26(3), 365‐373.

Lee, W. P., Wu, P. Y., Shih, W. M., Lee, M. Y., & Ho, L. H. (2015). The effectiveness of the newly designed thermal gown on hypothermic patients after spinal surgery [Randomized Controlled Trial]. Journal of Clinical Nursing, 24(19-20), 2779-2787.

Leeth, D., Mamaril, M., Oman, K. S., & Krumbach, B. (2010). Normothermia and patient comfort: a comparative study in an outpatient surgery setting [Comparative Study

Randomized Controlled Trial]. Journal of PeriAnesthesia Nursing, 25(3), 146-151.

Lenhardt, R., Marker, E., Goll, V., Tschernich, H., Kurz, A., Sessler, D. I., Narzt, E., & Lackner, F. (1997). Mild intraoperative hypothermia prolongs postanesthetic recovery [Clinical Trial

Comparative Study Randomized Controlled Trial Research Support, Non-U.S. Gov't Research Support, U.S. Gov't, P.H.S.]. Anesthesiology, 87(6), 1318-1323.

Leung, K. K., Lai, A., & Wu, A. (2007). A randomised controlled trial of the electric heating pad vs forced-air warming for preventing hypothermia during laparotomy [Comparative Study Randomized Controlled Trial]. Anaesthesia, 62(6), 605-608.

Liang, D. D., Shan, Y. L., & Wang, L. L. (2020). The effect of prophylactic rewarming on postoperative nausea and vomiting among patients undergoing laparoscopic hysterectomy: a prospective randomized clinical study. Sao Paulo Medical Journal, 138(5), 414-421.

Lindwall, R., Svensson, H., Söderström, S., & Blomqvist, H. (1998). Forced air warming and intraoperative hypothermia [Article]. European Journal of Surgery, 164(1), 13-16.

Luo, J. W., Zhou, L., Lin, S. M., Yan, W. C., Huang, L. J., & Liang, S. H. (2020). Beneficial effect of fluid warming in elderly patients with bladder cancer undergoing Da Vinci robotic-assisted laparoscopic radical cystectomy. Clinics, 75.

Ma, H., Lai, B., Dong, S., Li, X., Cui, Y., Sun, Q., Liu, W., Jiang, W., Xu, F., Lv, H., & et al. (2017). Warming infusion improves perioperative outcomes of elderly patients who underwent bilateral hip replacement [Journal Article; Randomized Controlled Trial]. Medicine, 96(13), e6490.

Macouillard, G., Cacchini, J., & Gadrat, F. (1995). Evaluation of three convective warming systems for prevention of hypothermia in major spinal surgery. British journal of anaesthesia european journal of anaesthesiologists annal congress, 74Supp117.

Maleki, A., Soltani, A. E., Goudarzi, M., Esbahbodi, E., Takzare, A., Zadeh, A. T., & Moadabi, M. (2018). Assessing the effect of warming up the patient with forced air on the body central temperature during general anesthesia in patients aged 20-70 years under eye surgery in Farabi Hospital. WORLD FAMILY MEDICINE, 16(1), 48-54.

Manwaring, J. M., Readman, E., & Maher, P. J. (2008). The effect of heated humidified carbon dioxide on postoperative pain, core temperature, and recovery times in patients having laparoscopic surgery: a randomized controlled trial [Randomized Controlled Trial]. Journal of Minimally Invasive Gynecology, 15(2), 161-165.

Maria, P. L. S., Maria, C. S., Eisenried, A., Velasquez, N., Kannard, B. T., Ramani, A., Kahn, D. M., Wheeler, A. J., & Brock-Utne, J. G. (2017). A novel thermal compression device for perioperative warming: A randomized trial for feasibility and efficacy [Article]. BMC Anesthesiology, 17(1).

Mason, D. S., Sapala, J. A., Wood, M. H., & Sapala, M. A. (1998). Influence of a forced air warming system on morbidly obese patients undergoing Roux-en-Y gastric bypass [Article]. Obesity Surgery, 8(4), 453-460.

Matsukawa, T., Kashimoto, S., Nakamura, T., Kume, M., Kanda, F., & Kumazawa, T. (1994). Effects of a forced-air system (Bair Hugger, OR-type) on intraoperative temperature in patients with open abdominal surgery [Article]. Journal of Anesthesia, 8(1), 25-27.

Matsuzaki, Y., Matsukawa, T., Ohki, K., Yamamoto, Y., Nakamura, M., & Oshibuchi, T. (2003). Warming by resistive heating maintains perioperative normothermia as well as forced air heating [Clinical Trial Randomized Controlled Trial]. British Journal of Anaesthesia, 90(5), 689-691.

Mecke, H., & Kroll, K. (1999). Does insufflation with warmed carbon dioxide reduce pain after laparoscopy? A randomized trial [Article]. Geburtshilfe und Frauenheilkunde, 59(12), 611-615.

Melling, A. C., Ali, B., Scott, E. M., & Leaper, D. J. (2001). Effects of preoperative warming on the incidence of wound infection after clean surgery: a randomised controlled trial [Clinical Trial Randomized Controlled Trial Research Support, Non-U.S. Gov't]. Lancet, 358(9285), 876-880.

Mihalik, H. A., & Robins, C. (2008). The effects of pre-warming on intraoperative and postoperative temperatures. AANA Journal, 76(5), 383-383.

Min, S. H., Yoon, S., Yoon, S. H., Bahk, J. H., & Seo, J. H. (2018). Randomised trial comparing forced-air warming to the upper or lower body to prevent hypothermia during thoracoscopic surgery in the lateral decubitus position [Comparative Study Randomized Controlled Trial]. British Journal of Anaesthesia, 120(3), 555-562.

Mogera, H., Dash, H. H., Chaturvedi, A., Tewari, R., & Bhutara, S. (1997). Control of body temperature with forced-air warming system during neurosurgery [Journal: Article]. Journal of anaesthesiology, clinical pharmacology, 13(3), 207‐212.

Monga, M., Comeaux, B., & Roberts, J. A. (1996). Effect of irrigating fluid on perioperative temperature regulation during transurethral prostatectomy [Clinical Trial; Journal Article; Randomized Controlled Trial]. European Urology, 29(1), 26‐28.

Monteiro, F. L. J., Halpern, H., Bortoli, F., Kataoka, L., Marumo, C., Ribeiro, M., Castellassi, M., Mendes, C., & Wolosker, N. (2017). Forced-Air Warming in Patients Undergoing Endovascular Procedures: comparison between 2 Thermal Blanket Models [Article In Press]. Annals of Vascular Surgery, (no pagination).

Motamed, C., Labaille, T., Léon, O., Panzani, J. P., Duvaldestin, P., & Benhamou, D. (2000). Core and thenar skin temperature variation during prolonged abdominal surgery: Comparison of two sites of active forced air warming [Article]. Acta Anaesthesiologica Scandinavica, 44(3), 249-254.

Motamed, S., Klubien, K., Edwardes, M., Mazza, L., & Carli, F. (1998). Metabolic changes during recovery in normothermic versus hypothermic patients undergoing surgery and receiving general anesthesia and epidural local anesthetic agents [Clinical Trial Comparative Study Randomized Controlled Trial Research Support, Non-U.S. Gov't]. Anesthesiology, 88(5), 1211-1218.

Mouton, W. G., Bessell, J. R., Millard, S. H., Baxter, P. S., & Maddern, G. J. (1999). A randomized controlled trial assessing the benefit of humidified insufflation gas during laparoscopic surgery [Clinical Trial Randomized Controlled Trial]. Surgical Endoscopy, 13(2), 106-108.

Moysés, A. M., Santos Trettene, A. d., Camacho Navarro, L. H., & Ayres, J. A. (2014). Hypothermia prevention during surgery: comparison between thermal mattress and thermal blanket. Revista da Escola de Enfermagem da USP, 48(2), 226-252.

Müller, C. M., Gabriel, A., Langenecker, S., Hartmann, T., Steltzer, H., Werba, A., Mühlbacher, F., & Zimpfer, M. (1993). Effectiveness of rapid infusion and Bair Hugger systems in maintaining normothermia during orthotopic liver transplantation [Clinical Trial; Comparative Study; Journal Article; Randomized Controlled Trial; Research Support, Non‐U.S. Gov't]. Transplantation Proceedings, 25(2), 1833‐1834.

Muller, C. M., Langenecker, S., Andel, H., Nantschev, I., Holzenbein, T. J., & Zimpfer, M. (1995). Forced-air warming maintains normothermia during orthotopic liver transplantation [Clinical Trial Randomized Controlled Trial Research Support, Non-U.S. Gov't]. Anaesthesia, 50(3), 229-232.

Munday, J., Osborne, S., Yates, P., Sturgess, D., Jones, L., & Gosden, E. (2018). Preoperative Warming Versus no Preoperative Warming for Maintenance of Normothermia in Women Receiving Intrathecal Morphine for Cesarean Delivery: A Single-Blinded, Randomized Controlled Trial [Comparative Study Randomized Controlled Trial Research Support, Non-U.S. Gov't]. Anesthesia & Analgesia, 126(1), 183-189.

Muth, C. M., Mainzer, B., & Peters, J. (1996). The use of countercurrent heat exchangers diminishes accidental hypothermia during abdominal aortic aneurysm surgery [Article]. Acta Anaesthesiologica Scandinavica, 40(10), 1197-1202.

NCT00651898. (2008a). Circulating-water Garment with Forced-air Warming and Circulating-water Mattress During Abdominal Surgery.

NCT00772460. (2008b). Comparison of a New Patient Warming System Using Polymer Conductive Warming with Forced Air Warming During Surgery.

NCT00711867. (2008c). Comparison of Intraoperative Warming Devices.

NCT00815191. (2008d). Comparison of Vital HEAT (vH2) Temperature Management System to Upper-body Forced-air Warming.

NCT00670826. (2008e). Comparison Study of Intraoperative Patient Warming Systems.

NCT00712023. (2008f). Effect of Forced-air Warming and Circulating-water Mattress in Preventing Heat Loss During Vascular Surgery.

NCT00642005. (2008g). Humidification in Laparoscopic Colonic Surgery.

NCT00617136. (2008h). Perioperative Temperature Management.

NCT00801424. (2008i). Warmed Humidified Carbon Dioxide (CO2) for Open Surgery.

NCT01234233. (2010a). Effects of Preoperative Warming of Patients on Postoperative Hypothermia and Shivering.

NCT01213628. (2010b). Local CO2 Increases Core and Wound Temperature.

NCT01056991. (2010c). Prevention of IPH: Electric Warming Mattress vs Forced Air Warming Blanket.

NCT01094119. (2010d). A Randomized Comparison of Intraoperative Warming with the LMA PerfecTemp and Forced-air.

NCT01054209. (2010e). A Study to Determine the Effectiveness of a Warming Mattress in Preventing Inadvertent Peri-operative Hypothermia and Shivering in Patients Undergoing Elective Cesarean Section.

NCT01285206. (2011a). Carbon Polymer Blankets to Prevent Incidence of Peri-Operative Hypothermia (IPH) in the DSU.

NCT01502163. (2011b). Efficacy of a Convective Prewarming System in Prevention of Perioperative Hypothermia.

NCT01649596. (2012). Impact of Expanded Peri Operative Warming.

NCT01795482. (2013a). Preoperative Patient Warming for Prevention of Perioperative Hypothermia in Major Abdominal Surgery.

NCT01900067. (2013b). Study to Evaluate Safety and Efficacy of Blanket Used to Prevent Anesthesia Induced Hypothermia.

NCT02201095. (2014a). Active Warming During Elective Caesearean Section.

NCT02214524. (2014b). Comparison of Active and Passive Perioperative Warming Techniques in Reducing Intraoperative Blood Loss.

NCT02079311. (2014c). Evaluation of Core Body Temperature When Using Forced Air Warming or an Active Blanket to Prevent Perioperative Hypothermia.

NCT02091466. (2014d). Pre-warming Prevents Hypothermia in Elective Cesarean Section.

NCT02243462. (2014e). Preoperative Warming and Perioperative Shivering.

NCT02177903. (2014f). Study to Determine the Effects of Pre-warming on OR Patient Temperatures and Surgical Outcomes.

NCT02467777. (2015a). Comparison of Forced Air and Conductive Patient Heating Systems During Ambulatory Surgeries.

NCT02586974. (2015b). Effects of Warmed, Humidified CO2 Insufflation on Body Core Temperature and Cytokine Response.

NCT02422758. (2015c). Prewarming Effect in Preventing Perioperative Hypothermia.

NCT02336152. (2015d). Use of Onepiece Suit or Forced Warm Air for Perioperative Temperature Conservation.

NCT02993666. (2016a). Comparison of Upper and Lower Body Air Warming in Patients Undergoing Thoracic Surgery.

NCT02715076. (2016b). The Effects of Ambient Temperature and Forced-air Warming on Intraoperative Core Temperature.

NCT02742818. (2016c). Forced-air Warming in Endovascular Surgery: Testing Effectiveness of Two Different Blanket Models.

NCT02990429. (2016d). Forced Air and Intravenous Fluid Warmers in Gynecologic Laparoscopic Surgery.

NCT02781194. (2016e). Temperature and Pain in Laparoscopy.

NCT03184506. (2017a). Effect of Pre-warming on Perioperative Hypothermia During HoLEPunder Spinal Anesthesia.

NCT03111875. (2017b). Perioperative Hypothermia and Myocardial Injury After Non-cardiac Surgery.

NCT03473470. (2018a). Evaluation of the Active Warming Effects on Maternal and Neonatal Outcome During Cesarean Delivery.

NCT03630887. (2018b). Prevention of Perioperative Hypothermia in Patients Submitted to Transurethral Resection.

NCT03420924. (2018c). Thermal Suit with Forced-air Warming in Breast Cancer Surgery.

NCT03824262. (2019a). The Comparison of Warming Techniques in Patients Undergoing Open Abdominal Surgery in Litotomy Position.

NCT04027842. (2019b). Effect of 10 Minute-prewarming on Core Body Temperature During Gynecologic Laparoscopic Surgery Under General Anesthesia.

NCT04164706. (2019c). HEAT HumiGard Evaluation Study.

NCT04244071. (2020). The Effect of Heating on Thermal Comfort and Anxiety.

NCT04741815. (2021). Effects of Different Warming Methods in Laparoscopic Cholecystectomy Surgery.

NCT05334589. (2022). The Effect of Preoperative Active Warming on Intraoperative Body Temperature.

Negishi, C., Hasegawa, K., Mukai, S., Nakagawa, F., Ozaki, M., & Sessler, D. I. (2003). Resistive-heating and forced-air warming are comparably effective [Clinical Trial Comparative Study Randomized Controlled Trial Research Support, Non-U.S. Gov't Research Support, U.S. Gov't, P.H.S.]. Anesthesia & Analgesia, 96(6), 1683-1687.

Nelskylä, K., Yli-Hankala, A., Sjöberg, J., Korhonen, I., & Korttila, K. (1999). Warming of insufflation gas during laparoscopic hysterectomy: Effect on body temperature and the autonomic nervous system [Article]. Acta Anaesthesiologica Scandinavica, 43(10), 974-978.

Ng, S. F., Oo, C. S., Loh, K. H., Lim, P. Y., Chan, Y. H., & Ong, B. C. (2003). A comparative study of three warming interventions to determine the most effective in maintaining perioperative normothermia [Article]. Anesthesia and Analgesia, 96(1), 171-176.

Ng, V., Lai, A., & Ho, V. (2006). Comparison of forced-air warming and electric heating pad for maintenance of body temperature during total knee replacement [Article]. Anaesthesia, 61(11), 1100-1104.

Nguyen, H. P., Zaroff, J. G., Bayman, E. O., Gelb, A. W., Todd, M. M., & Hindman, B. J. (2010). Perioperative hypothermia (33 degrees C) does not increase the occurrence of cardiovascular events in patients undergoing cerebral aneurysm surgery: findings from the Intraoperative Hypothermia for Aneurysm Surgery Trial [Clinical Trial; Comparative Study; Journal Article; Randomized Controlled Trial; Research Support, N.I.H., Extramural; Research Support, Non‐U.S. Gov't]. Anesthesiology, 113(2), 327‐342.

Nguyen, N. T., Furdui, G., Fleming, N. W., Lee, S. J., Goldman, C. D., Singh, A., & Wolfe, B. M. (2002). Effect of heated and humidified carbon dioxide gas on core temperature and postoperative pain - A randomized trial. Surgical Endoscopy and Other Interventional Techniques, 16(7), 1050-1054.

NTR6495. (2017). Prevention of hypothermia in primary joint replacement patients comparing a conductive blanket with a hot forced air system.

O'Brien, D., Greenfield, M. L. V. H., Anderson, J. E., Smith, B. A., & Morris, M. (2010). Comfort, Satisfaction, and Anxiolysis in Surgical Patients Using a Patient-Adjustable Comfort Warming System: A Prospective Randomized Clinical Trial [Article]. Journal of PeriAnesthesia Nursing, 25(2), 88-93.

Ochampaugh, B. U., & Glenning, C. (2011). The Effects of Forced Air Warming in Preventing Post-Operative Hypothermia. Journal of PeriAnesthesia Nursing, 26(3), 201-201.

Oderda, M., Cerutti, E., Gontero, P., Manetta, T., Mengozzi, G., Meyer, N., Munegato, S., Noll, E., Rampa, P., Piechaud, T., & Diemunsch, P. (2018). Effects of warmed, humidified CO2 insufflation on body core temperature and cytokine response: head-to-head randomized comparison vs. standard insufflation during RARP. Minerva Anestesiologica, 84(10), 1228-1230.

Oderda, M., Cerutti, E., Gontero, P., Manetta, T., Mengozzi, G., Meyer, N., Munegato, S., Noll, E., Rampa, P., Piéchaud, T., & Diemunsch, P. (2019). The impact of warmed and humidified CO2 insufflation during robotic radical prostatectomy: Results of a randomized controlled trial [Article]. Urologia Journal, 86(3), 130-140.

Oh, J. H., Kim, J. Y., Chung, S. W., Park, J. S., Kim, D. H., Kim, S. H., & Yun, M. J. (2014). Warmed irrigation fluid does not decrease perioperative hypothermia during arthroscopic shoulder surgery [Article]. Arthroscopy - Journal of Arthroscopic and Related Surgery, 30(2), 159-164.

Ohki, K., Kawano, R., Yoshida, M., Kanosue, I., & Yamamoto, K. (2019). Normothermia is Best Achieved by Warming Above and Below with Pre-warming Adjunct: a Comparison of Conductive Fabric Versus Forced-air and Water [Comparative Study; Journal Article; Randomized Controlled Trial]. Surgical technology international, 34, 40‐45.

Okada, N., Fujita, T., Kanamori, J., Sato, A., Kurita, D., Horikiri, Y., Sato, T., Fujiwara, H., Yamamoto, H., & Daiko, H. (2020). Efficacy of prewarming prophylaxis method for intraoperative hypothermia during thoracoscopic esophagectomy [Article]. Esophagus, 17(4), 385-391.

Okeke, L. I. (2007). Effect of warm intravenous and irrigating fluids on body temperature during transurethral resection of the prostate gland [Comparative Study Randomized Controlled Trial]. BMC Urology, 7, 15.

Omar, M., Monga, M., Noble, M., & Sivalingam, S. (2018). Hypothermia after percutaneous nephrolithotomy: A randomized single-blind clinical trial evaluating impact of irrigation fluid temperature [Conference Abstract]. Journal of Urology, 199(4), e888.

Ong, B., Benson, E., & McMillan, D. (2012). Patient controlled warming improves outcome after knee arthroplasty [Conference Abstract]. Canadian Journal of Anesthesia, 59.

Ott, D. E., Reich, H., Love, B., McCorvey, R., Toledo, A., Liu, C. Y., Syed, R., & Kumar, K. (1998). Reduction of laparoscopic-induced hypothermia, postoperative pain and recovery room length of stay by pre-conditioning gas with the Insuflow device: a prospective randomized controlled multi-center study [Article]. JSLS : Journal of the Society of Laparoendoscopic Surgeons / Society of Laparoendoscopic Surgeons, 2(4), 321-329.

Ouuellette, R. G. (1993). Comparison of four intraoperative warming devices. AANA Journal, 61(4), 394-396.

Ozgonul, A., Erkan, C., Mehmet, G., Zeynep, B., & Ali, U. (2007). The effects of isothermic or hypothermic carbondioxide pneumoperitoneum on arterial blood gases. Saudi Medical Journal, 28(11), 1662-1665.

Pagnocca, M. L., Tai, E. J., & Dwan, J. L. (2009). Temperature control in conventional abdominal surgery: comparison between conductive and the association of conductive and convective warming [Randomized Controlled Trial]. Revista Brasileira de Anestesiologia, 59(1), 56-66.

Pan, X. Y., Ye, L. Y., Liu, Z. T., Wen, H., Hu, Y. Z., & Xu, X. X. (2015). Effect of irrigation fluid temperature on core body temperature and inflammatory response during arthroscopic shoulder surgery. Archives of Orthopaedic and Trauma Surgery, 135(8), 1131-1139.

Paris, L. G., Seitz, M., McElroy, K. G., & Regan, M. (2014). A randomized controlled trial to improve outcomes utilizing various warming techniques during cesarean birth [Randomized Controlled Trial]. JOGNN - Journal of Obstetric, Gynecologic, & Neonatal Nursing, 43(6), 719-728.

Park, H. J., Moon, H. S., Moon, S. H., Jeong, H. D., Jeon, Y. J., Han, K. D., & Koh, H. J. (2017). The effect of humidified heated breathing circuit on core body temperature in perioperative hypothermia during thyroid surgery [Article]. International Journal of Medical Sciences, 14(8), 791-797.

Park, O. B., & Choi, H. (2010). [The effect of pre-warming for patients under abdominal surgery on body temperature, anxiety, pain, and thermal comfort] [Controlled Clinical Trial]. Journal of Korean Academy of Nursing, 40(3), 317-325.

Park, S., Yoon, S. H., Youn, A. M., Song, S. H., & Hwang, J. G. (2017). Heated wire humidification circuit attenuates the decrease of core temperature during general anesthesia in patients undergoing arthroscopic hip surgery [Article]. Korean Journal of Anesthesiology, 70(6), 619-625.

Pearce, B., Mattheyse, L., Ellard, L., Desmond, F., Pillai, P., & Weinberg, L. (2018). Comparison of the WarmCloud and Bair Hugger Warming Devices for the Prevention of Intraoperative Hypothermia in Patients Undergoing Orthotopic Liver Transplantation: A Randomized Clinical Trial. TRANSPLANTATION DIRECT, 4(4).

Pei, L. J., Huang, Y. G., Xu, Y. Y., Zheng, Y. C., Sang, X. T., Zhou, X. Y., Li, S. Q., Mao, G. M., Mascha, E. J., & Sessler, D. I. (2018). Effects of Ambient Temperature and Forced- air Warming on Intraoperative Core Temperature A Factorial Randomized Trial. Anesthesiology, 128(5), 903-911.

Peña, I., & García, M. (1996). Risk of hypothermia in prolonged anesthesia and surgery. Study of two warming methods. Revista ROL de enfermería, 3(211), 25‐31.

Perez-Protto, S., Sessler, D. I., Reynolds, L. F., Bakri, M. H., Mascha, E., Cywinski, J., Parker, B., Argalious, M., Perez-Protto, S., Sessler, D. I., Reynolds, L. F., Bakri, M. H., Mascha, E., Cywinski, J., Parker, B., & Argalious, M. (2010). Circulating-water garment or the combination of a circulating-water mattress and forced-air cover to maintain core temperature during major upper-abdominal surgery. BJA: The British Journal of Anaesthesia, 105(4), 466-470.

Perl, T., Peichl, L. H., Reyntjens, K., Deblaere, I., Zaballos, J. M., & Brauer, A. (2014). Efficacy of a novel prewarming system in the prevention of perioperative hypothermia. A prospective, randomized, multicenter study [Multicenter Study Randomized Controlled Trial]. Minerva Anestesiologica, 80(4), 436-443.

Perl, T., Rhenius, A., Eich, C. B., Quintel, M., Heise, D., & Bräuer, A. (2012). Conductive warming and insulation reduces perioperative hypothermia [Article]. Central European Journal of Medicine, 7(3), 284-289.

Persson, K., & Lundberg, J. (2001). Perioperative hypothermia and postoperative opioid requirements [Article]. European Journal of Anaesthesiology, 18(10), 679-686.

Pit, M. J., Tegelaar, R. J., & Venema, P. L. (1996). Isothermic irrigation during transurethral resection of the prostate: Effects on peri-operative hypothermia, blood loss, resection time and patient satisfaction [Article]. British Journal of Urology, 78(1), 99-103.

Pu, Y., Cen, G., Sun, J., Gong, J., Zhang, Y., Zhang, M., Wu, X., Zhang, J., Qiu, Z., & Fang, F. (2014). Warming with an underbody warming system reduces intraoperative hypothermia in patients undergoing laparoscopic gastrointestinal surgery: A randomized controlled study [Article]. International Journal of Nursing Studies, 51(2), 181-189.

Puttick, M. I., Scott-Coombes, D. M., Dye, J., Nduka, C. C., Menzies-Gow, N. M., Mansfield, A. O., & Darzi, A. (1999). Comparison of immunologic and physiologic effects of CO2 pneumoperitoneum at room and body temperatures [Article]. Surgical Endoscopy, 13(6), 572-575.

Ralte, P., Mateu-Torres, F., Winton, J., Bardsley, J., Smith, M., Kent, M., Sethuraman, D., & Guisasola, I. (2020). Prevention of Perioperative Hypothermia: A Prospective, Randomized, Controlled Trial of Bair Hugger Versus Inditherm in Patients Undergoing Elective Arthroscopic Shoulder Surgery [Randomized Controlled Trial]. Arthroscopy, 36(2), 347-352.

Ramsay, R., Sampurno, S., Chittleborough, T., Hiller, J., Warrier, S., Heriot, A., & Lynch, C. (2022). Surgical humidification impacts local and systemic inflammation and peritoneal trauma in colorectal cancer surgery: a randomised controlled trial [Journal: Conference Abstract]. Colorectal Disease, 24(SUPPL 1), 5‐6.

Rathinam, S., Annam, V., Steyn, R., & Raghuraman, G. (2009). A randomised controlled trial comparing Mediwrap® heat retention and forced air warming for maintaining normothermia in thoracic surgery [Conference Paper]. Interactive Cardiovascular and Thoracic Surgery, 9(1), 15-19.

Ravindran, S., Aloysius, L., Balakrishnan, M., & Krishna, K. M. J. (2021). Effect of preoperative warming on post induction core temperature and incidence of postoperative shivering in patients undergoing general anaesthesia [Conference Abstract]. Anesthesia and Analgesia, 133(3 SUPPL 2), 1615-1616.

Reeves, N., White, J., Bird, S., Shinkwin, M., Cornish, J., & Torkington, J. (2021). Warmed and humidified insufflation to prevent perioperative hypothermia and improve the quality of recovery in elective laparoscopic colorectal resection patients: a feasibility study for a triple-blind randomized controlled trial [Article]. Colorectal Disease, 23(12), 3262-3271.

Reeves, N., White, J., Shinkwin, M., Ansell, J., Horwood, J., Cornish, J., & Torkington, J. (2021). Heat: Quality of Recovery and Perioperative Hypothermia in Elective Colectomy Patients. A Feasibility Study of a Blinded Randomised Controlled Trial [Conference Abstract]. Colorectal Disease, 23(SUPPL 1), 102.

Rein, E. B., Filtvedt, M., Walløe, L., & Ræder, J. C. (2007). Hypothermia during laparotomy can be prevented by locally applied warm water and pulsating negative pressure [Article]. British Journal of Anaesthesia, 98(3), 331-336.

Ruetzler, K., Kovaci, B., Guloglu, E., Kabon, B., Fleischmann, E., Kurz, A., Mascha, E., Dietz, D., Remzi, F., & Sessler, D. I. (2011). Forced-air and a novel patient-warming system (vitalHEAT vH2) comparably maintain normothermia during open abdominal surgery [Comparative Study Randomized Controlled Trial Research Support, Non-U.S. Gov't]. Anesthesia & Analgesia, 112(3), 608-614.

Rui, Z., Jiawei, Q., Jinlin, W., Wenxiang, J., Enzehua, X., Wei, G., Cuntao, Y., & Juntao, Q. (2020). Effect of heated humidified ventilation on intraoperative core temperature and prognosis in normothermic thoraco-abdominal aortic aneurysm repair. Journal of Thoracic Disease, 12(3), 276‐283.

Rui, Z., Xueli, C., Yan, X., Zhang, R., Chen, X., & Xiao, Y. (2018). The effects of a forced-air warming system plus electric blanket for elderly patients undergoing transurethral resection of the prostate: A randomized controlled trial. Medicine, 97(45), 1-7.

Russell, S. H., & Freeman, J. W. (1995). Prevention of hypothermia during orthotopic liver transplantation: comparison of three different intraoperative warming methods [Clinical Trial; Journal Article; Randomized Controlled Trial]. British Journal of Anaesthesia, 74(4), 415‐418.

Saad, S., Minor, I., Mohri, T., & Nagelschmidt, M. (2000). The clinical impact of warmed insufflation carbon dioxide gas for laparoscopic cholecystectomy [Article]. Surgical Endoscopy, 14(9), 787-790.

Salazar, C. A., Wong, M. C., Miller, V. E., Morris, S. N., & Isaacson, K. B. (2019). The Effect of Warmed Hysteroscopic Fluid-Distention Medium on Postoperative Core Body Temperature: A Randomized Trial [Article]. Journal of Gynecologic Surgery, 35(4), 239-245.

Salazar, C. A., Wong, M. C., Morris, S. N., & Isaacson, K. B. (2017). The effect of warmed hysteroscopic fluid distention medium on postoperative core body temperature: A randomized control trial [Conference Abstract]. Journal of Minimally Invasive Gynecology, 24(7), S142.

Salazar, F., Doñte, M., Boget, T., Bogdanovich, A., Basora, M., Torres, F., & Fàbregas, N. (2011). Intraoperative warming and post-operative cognitive dysfunction after total knee replacement [Article]. Acta Anaesthesiologica Scandinavica, 55(2), 216-222.

Sammour, T., & Hill, A. G. (2015). Five Year Follow-Up of a Randomized Controlled Trial on Warming and Humidification of Insufflation Gas in Laparoscopic Colonic Surgery-Impact on Small Bowel Obstruction and Oncologic Outcomes. International Surgery, 100(4), 608-616.

Sammour, T., Kahokehr, A., Hayes, J., Hulme-Moir, M., & Hill, A. G. (2010). Warming and humidification of insufflation carbon dioxide in laparoscopic colonic surgery: a double-blinded randomized controlled trial [Multicenter Study Randomized Controlled Trial Research Support, Non-U.S. Gov't]. Annals of Surgery, 251(6), 1024-1033.

Sammour, T., Kahokehr, A., Hayes, J., Hulme Moir, M., & Hill, A. G. (2010). Warming and humidification of insufflation CO2 in laparoscopic colonic surgery - A double-blinded randomised controlled trial [Conference Abstract]. Journal of Surgical Research, 158(2), 174.

Santos, R. M. D. S. F., Boin, I. F. S. F., Caruy, C. A. A., Cintra, E. A., Torres, N. A., & Duarte, H. N. (2019). Randomized clinical study comparing active heating methods for prevention of intraoperative hypothermia in gastroenterology [Article]. Revista Latino-Americana de Enfermagem, 27, e3103.

Savel, R. H., Balasubramanya, S., Lasheen, S., Gaprindashvili, T., Arabov, E., Fazylov, R. M., Lazzaro, R. S., & Macura, J. M. (2005). Beneficial effects of humidified, warmed carbon dioxide insufflation during laparoscopic bariatric surgery: A randomized clinical trial [Article]. Obesity Surgery, 15(1), 64-69.

Schmied, H., Kurz, A., Sessler, D. I., Kozek, S., & Reiter, A. (1996). Mild hypothermia increases blood loss and transfusion requirements during total hip arthroplasty [Article]. Lancet, 347(8997), 289-292.

Schroeder, F., Horn, E. P., Redmann, G., & Standl, T. (1999). Partial body heating preserves normothermia in patients undergoing orthopaedic surgery [Article]. Anasthesiologie Intensivmedizin Notfallmedizin Schmerztherapie, 34(8), 475-479.

Schroeder, K. M., Riley, S., Chambers, T., & Shepler, J. (2015). Impact of preoperative forced-air warming on perioperative thermoregulation in patients undergoing total hip arthroplasty under neuraxial anesthesia [Conference Abstract]. Regional Anesthesia and Pain Medicine, 40(5).

Scott, E. M. (2007). Systemic warming before, during, and after major abdominal surgery reduced postoperative complications more than warming during surgery only. Evidence Based Nursing, 10(4), 114-114.

Scott, E. M., Leaper, D. J., Clark, M., & Kelly, P. J. (2001). Effects of warming therapy on pressure ulcers--a randomized trial [Case Reports Clinical Trial Randomized Controlled Trial Review]. AORN Journal, 73(5), 921-927, 929-933, 936-928.

Sethuraman, D., Guisasola, I., & Mateu-Torres, F. (2021). A comparative study of Bair Hugger® vs. Inditherm® in arthroscopic shoulder surgery [Conference Abstract]. Anaesthesia, 76(SUPPL 6), 19.

Shao, L., Pang, N. N., Yan, P., Jia, F. J., Sun, Q., Ma, W. J., & Yang, Y. (2018). Control of body temperature and immune function in patients undergoing open surgery for gastric cancer. Bosnian Journal of Basic Medical Sciences, 18(3), 289-296.

Shao, L., Zheng, H., Jia, F. J., Wang, H. Q., Liu, L., Sun, Q., An, M. Y., Zhang, X. H., & Wen, H. (2012). Methods of patient warming during abdominal surgery [Article]. PLoS ONE, 7(7).

Shariffuddin, II, Hasan, M. S., Chong, T. H., Kwan, M. K., & Chan, Y. K. (2016). Under-body forced-air warming blanket versus resistive heating blanket for prevention of hypothermia during spinal surgery: a randomized prospective study [Journal: Article]. Journal of Health and Translational Medicine, 19(1).

Sharma, M. P. (2012). A study to determine whether carbon polymer warming blankets can reduce the incidence of inadvertent peri-operative hypothermia (iph) during day-case surgery [Conference Abstract]. British Journal of Anaesthesia, 108, ii345-ii346.

Shereen Tang Suet, P., Tai Li, L., Esa, K., Mohamad, M., & Jaafar Md, Z. (2015). Forced Air Warming during Hysterectomy under Combined Epidural and General Anaesthesia: Comparison of Upper with Lower Body Warming. International Medical Journal, 22(4), 295-298.

Shin, K. M., Ahn, J. H., Kim, I. S., Lee, J. Y., Kang, S. S., Hong, S. J., Chung, H. M., & Lee, H. J. (2015). The efficacy of pre-warming on reducing intraprocedural hypothermia in endovascular coiling of cerebral aneurysms [Article]. BMC Anesthesiology, 15(1).

RBR-52shjp. (2018). Prevention of body temperature drop during surgery, using different models of heaters.

Si, J. L., Yang, M. Q., Zhang, L. Y., Sima, L. J., & Dong, X. (2017). Effect of perioperative temperature protection on the coagulation function during total knee arthroplasty [Journal: Article]. Chinese Journal of Tissue Engineering Research, 21(23), 3652‐3657.

Siddiqui, J., Killick, S., Doto, T., Lindow, S., & Phillips, K. (2009). Heating and hydrating the insufflating gas at laparoscopy: Double-blind, prospective, randomised controlled trial - Pilot study [Conference Abstract]. Gynecological Surgery, 6, S140.

Slim, K., Bousquet, J., Kwiatkowski, F., Lescure, G., Pezet, D., & Chipponi, J. (1999). Effect of CO(2) gas warming on pain after laparoscopic surgery: a randomized double-blind controlled trial [Clinical Trial Randomized Controlled Trial]. Surgical Endoscopy, 13(11), 1110-1114.

Smith, C. E., Desai, R., Glorioso, V., Cooper, A., Pinchak, A. C., & Hagen, J. F. (1998). Preventing hypothermia: Convective and intravenous fluid warming versus convective warming alone [Article]. Journal of Clinical Anesthesia, 10(5), 380-385.

Smith, C. E., Gerdes, E., Sweda, S., Myles, C., Punjabi, A., Pinchak, A. C., & Hagen, J. F. (1998). Warming intravenous fluids reduces perioperative hypothermia in women undergoing ambulatory gynecological surgery [Article]. Anesthesia and Analgesia, 87(1), 37-41.

Smith, I., Newson, C. D., & White, P. F. (1994). Use of forced-air warming during and after outpatient arthroscopic surgery [Clinical Trial Randomized Controlled Trial]. Anesthesia & Analgesia, 78(5), 836-841.

So Young Lee, S. J. K., Jin-Yong Jung. (2020). Effects of 10-min prewarming on core body temperature during gynecologic laparoscopic surgery under general anesthesia: a randomized controlled trial [Clinical Research]. Anesthesiology and Pain Medicine, 15(3), 349‐355.

Song, X. (2010). Study on influence of temperature-variable circulating water blanket on body temperature of surgical patients. Chinese Nursing Research, 24(1A), 33-34.

Steib, A., Beller, J. P., Von Bandel, M., Beck, F., Chabrol, J. L., & Otteni, J. C. (1993). Oesophageal thermal tube for intraoperative hypothermia in liver transplantation [Article]. Acta Anaesthesiologica Scandinavica, 37(2), 199-202.

Steinbrook, R. A., & Seigne, P. W. (1997). Total-body oxygen consumption after isoflurane anesthesia: Effects of mild hypothermia and combined epidural general anesthesia. Journal of Clinical Anesthesia, 9(7), 559-563.

Stirparo, S., Laudani, A., & Capogna, G. (2011). Forced air warmer favors safe skin-to-skin contact (SSC) during cesarean section maintaining neonatal normothermia [Conference Abstract]. Regional Anesthesia and Pain Medicine, 36(5), E198-E199.

Su, S. F., & Nieh, H. C. (2018). Efficacy of forced-air warming for preventing perioperative hypothermia and related complications in patients undergoing laparoscopic surgery: A randomized controlled trial [Randomized Controlled Trial]. International Journal of Nursing Practice, 24(5), e12660.

Sukcharanjit, S., Tan, A. S. B., Loo, A. V. P., Chan, X. L., & Wang, C. Y. (2015). The effect of a forced-air warming blanket on patients' end-tidal and transcutaneous carbon dioxide partial pressures during eye surgery under local anaesthesia: A single-blind, randomised controlled trial [Article]. Anaesthesia, 70(12), 1390-1394.

Suraseranivongse, S., Pongraweewan, O., Kongmuang, B., Tivirach, W., & Pornboonseram, S. (2009). A custom-made forced-air warming mattress for heat loss prevention during vascular surgery: Clinical evaluation [Article]. Asian Biomedicine, 3(3), 299-307.

Sutton, E., Bellini, G., Kumara, H. S., Yan, X., Njoh, L., Cekic, V., & Whelan, R. L. (2016). Warm and humidified vs cold and dry CO2 pneumoperitoneum in minimally invasive colon resection: A randomized controlled trial [Conference Abstract]. Surgical Endoscopy and Other Interventional Techniques, 30, S317.

Tanaka, N., Ohno, Y., Hori, M., Utada, M., Ito, K., & Suzuki, T. (2013). A randomised controlled trial of the resistive heating blanket versus the convective warming system for preventing hypothermia during major abdominal surgery [Randomized Controlled Trial]. Journal of Perioperative Practice, 23(4), 82-86.

Tekgul, Z. T., Pektas, S., Yildirim, U., Karaman, Y., Cakmak, M., Ozkarakas, H., & Gonullu, M. (2014). A prospective randomized double-blind study on the effects of the temperature of irrigation solutions on thermoregulation and postoperative complications in percutaneous nephrolithotomy [Article in Press]. Journal of Anesthesia.

Teoh, T., Lim, S., Chaw, S., Yim, C., & Ishak, J. (2022). Does higher operation theatre temperature range reduce the incidence of post-spinal shivering in obstetric patients? A doubleblind, prospective, randomised control study [Conference Abstract]. Anaesthesia, 77(SUPPL 2), 53.

Thapa, H. P., Kerton, A. J., & Peyton, P. J. (2019). Comparison of the EasyWarm® self-heating blanket with the Cocoon forced-air warming blanket in preventing intraoperative hypothermia [Article]. Anaesthesia and Intensive Care, 47(2), 169-174.

Thiel, B., Mooijer, B. C., Kolff-Gart, A. S., Kerklaan, B. M., Poolman, R. W., de Haan, P., & Siepel, M. A. M. (2020). Is preoperative forced-air warming effective in the prevention of hypothermia in orthopedic surgical patients? A randomized controlled trial [Letter Randomized Controlled Trial]. Journal of Clinical Anesthesia, 61, 109633.

Thompson, H., Vandeleur, A., Agarwal, A., Hodgson, R., Appleyard, M., & Rahman, T. M. (2013). Hypothermia in endoscopy [Conference Abstract]. Journal of Gastroenterology and Hepatology, 28, 50.

Tjoakarfa, C., David, V., Ko, A., & Hau, R. (2017). Reflective Blankets Are as Effective as Forced Air Warmers in Maintaining Patient Normothermia During Hip and Knee Arthroplasty Surgery [Article]. Journal of Arthroplasty, 32(2), 624-627.

Torossian, A., Van Gerven, E., Geertsen, K., Horn, B., Van de Velde, M., & Raeder, J. (2016). Active perioperative patient warming using a self-warming blanket (BARRIER EasyWarm) is superior to passive thermal insulation: a multinational, multicenter, randomized trial [Multicenter Study Randomized Controlled Trial]. Journal of Clinical Anesthesia, 34, 547-554.

Torrie, J. J., Yip, P., & Robinson, E. (2005). Comparison of forced-air warming and radiant heating during transurethral prostatic resection under spinal anaesthesia [Comparative Study; Journal Article; Randomized Controlled Trial]. Anaesthesia and Intensive Care, 33(6), 733‐738.

Tramontini, C. C., & Graziano, K. U. (2007). Hypothermia control in elderly surgical patients in the intraoperative period: Evaluation of two nursing interventions. Revista Latino-Americana de Enfermagem, 15(4), 626-631.

Tramontini Fuganti, C. C., Zangiacomi Martinez, E., & Galvão, C. M. (2018). Effect of preheating on the maintenance of body temperature in surgical patients: a randomized clinical trial. Revista Latino-Americana de Enfermagem (RLAE), 26, 1-10.

Trentman, T. L., Weinmeister, K. P., Hentz, J. G., Laney, M. B., & Simula, D. V. (2009). Randomized non-inferiority trial of the vitalHEAT™ temperature management system vs the bair hugger® warmer during total knee arthroplasty [Article]. Canadian Journal of

Tuna, P. T., Kursun, S., & Kara, I. (2022). EFFECT OF ACTIVE AND PASSIVE HEATING METHODS USED IN DIFFERENT AREAS OF PERIOPERATIVE PROCESSES ON THERMAL COMFORT AND ANXIETY: A RANDOMIZED CONTROL TRIAL. JOURNAL OF BASIC AND CLINICAL HEALTH SCIENCES, 6(1), 225-237.

Tyvold, S. S. (2019). Preventing hypothermia in outpatient plastic surgery by self-warming or forced-air-warming blanket: A randomised controlled trial [Comparative Study Randomized Controlled Trial Research Support, Non-U.S. Gov't]. European Journal of Anaesthesiology, 36(11), 843-850.

Uys P., Soeters R., & Jeffery S. (2017). A randomised controlled trial of two methods to prevent hypothermia during major open gynaecological surgery [Conference Abstract]. BJOG International Journal of Obstetrics Gynaecology, 124, 85.

Vanni, S. M. D., Castiglia, Y. M. M., Ganem, E. M., Rodrigues Jr, G. R., Amorim, R. B., Ferrai, F., Braz, L. G., & Braz, J. R. C. (2007). Preoperative warming combined with intraoperative skin-surface warming does not avoid hypothermia caused by spinal anesthesia in patients with midazolam premedication [Article]. Sao Paulo Medical Journal, 125(3), 144-149.

Vanni, S. M. D., Cerqueira Braz, J. R., Pinheiro Módolo, N. S., Amorim, R. B., & Rodrigues Jr, G. R. (2003). Preoperative combined with intraoperative skin-surface warming avoids hypothermia caused by general anesthesia and surgery [Article]. Journal of Clinical Anesthesia, 15(2), 119-125.

Vecchio, J. J. D., Chemes, L. N., Ghioldi, M. E., Dealbera, E. D., & Daniel Morgillo, P. (2020). Comparison of two forced-air warming devices during foot and ankle surgery: a randomised controlled trial [Article]. Journal of Perioperative Practice, 30(11), 340-344.

Verra, W. C., Beekhuizen, S. R., van Kampen, P. M., de Jager, M. C., Deijkers, R. L. M., & Tordoir, R. L. (2018). Self-Warming Blanket Versus Forced-Air Warming in Primary Knee or Hip Replacement: A Randomized Controlled Non-Inferiority Study [Article]. Asian journal of anesthesiology, 56(4), 128-135.

Wagner, D., Byrne, M., & Kolcaba, K. (2006). Effects of comfort warming on preoperative patients. AORN Journal, 84(3), 427-436.

Wagner, K., Swanson, E., Raymond, C. J., & Smith, C. E. (2008). Comparison of two convective warming systems during major abdominal and orthopedic surgery [Article]. Canadian Journal of Anesthesia, 55(6), 358-363.

Wagner, V. D. (2007). Effect of a preoperative warming intervention on the acute phase response of surgical stress (Publication Number Ph.D.) University of South Florida]. CINAHL Plus with Full Text.

Wan Muhd Shukeri, W. F., Wan Hassan, W. M. N., & Mohamad Zaini, R. H. (2016). Passive Warming using a Heat-Band versus a Resistive Heating Blanket for the Prevention of Inadvertent Perioperative Hypothermia during Laparotomy for Gynaecological Surgery. Malaysian Journal of Medical Sciences, 23(2), 28-37.

Wang, Z. G., Chen, Z. Y., Kuang, R. X., Liu, S., Li, H. C., Zhang, W. N., Miao, Y. X., & Xu, Q. C. (2010). [Effect of the tumescent infiltration solution temperature on body temperature] [Randomized Controlled Trial]. Zhonghua Zheng Xing Wai Ke Za Zhi, 26(4), 269-272.

Wasfie, T. J., & Barber, K. R. (2015). Value of extended warming in patients undergoing elective surgery [Article]. International Surgery, 100(1), 105-108.

Wei, C., Yu, Y., Chen, Y., Wei, Y., & Ni, X. (2014). Impact of warming blood transfusion and infusion toward cerebral oxygen metabolism and cognitive recovery in the perioperative period of elderly knee replacement [Randomized Controlled Trial Research Support, Non-U.S. Gov't]. Journal of Orthopaedic Surgery, 9, 8.

Wei, L. J., Xu, P. J., & Qi, W. (2017). Effect of preoperative combined warming strategy on body temperature and recovery quality in patients undergoing major abdominal surgeries [Article]. World Chinese Journal of Digestology, 25(32), 2916-2920.

Weinberg, L., Alban, D., Jones, R., Story, D., Pearce, B., & McNicol, L. (2014). Prevention of hypothermia in patients undergoing orthotopic liver transplantation using the fisher and paykel humigard open surgery humidification system: A prospective randomised pilot clinical trial [Conference Abstract]. Liver Transplantation, 20, S154-S155.

Weinberg, L., Huang, A., Alban, D., Jones, R., Story, D., McNicol, L., & Pearce, B. (2017). Prevention of hypothermia in patients undergoing orthotopic liver transplantation using the humigard® open surgery humidification system: a prospective randomized pilot and feasibility clinical trial [Article]. BMC Surgery, 17(1), 10.

Whitney, A. (1990). The efficiency of a reflective heating blanket in preventing hypothermia in patients undergoing intra-abdominal procedures. AANA Journal, 58(3), 212-215.

Winkler, M., Akça, O., Birkenberg, B., Hetz, H., Scheck, T., Arkiliç, C. F., Kabon, B., Marker, E., Grübl, A., Czepan, R., & et al. (2000). Aggressive warming reduces blood loss during hip arthroplasty [Clinical Trial; Journal Article; Randomized Controlled Trial; Research Support, Non‐U.S. Gov't; Research Support, U.S. Gov't, P.H.S.]. Anesthesia and Analgesia, 91(4), 978‐984.

Wittenborn, J., Mathei, D., van Waesberghe, J., Zeppernick, F., Zeppernick, M., Tchaikovski, S., Kowark, A., Breuer, M., Keszei, A., Stickeler, E., Zoremba, N., Rossaint, R., Bruells, C., & Meinhold-Heerlein, I. (2022). The effect of warm and humidified gas insufflation in gynecological laparoscopy on maintenance of body temperature: a prospective randomized controlled multi-arm trial [Article in Press]. Archives of Gynecology and Obstetrics.

Wong, A., Walker, S., & Bradley, M. (2004). Comparison of a radiant patient warming device with forces aire warming during laparoscopic cholecystectomy [Article]. Anaesthesia and Intensive Care, 32(1), 93-99.

Wongprasartsuk, P., Konstantatos, A., & McRae, R. (1998). The effect of forced air warming on postoperative oxygen consumption and temperature in elective orthopaedic surgery [Article]. Anaesthesia and Intensive Care, 26(3), 267-271.

Woolnough, M., Allam, J., Hemingway, C., Cox, M., & Yentis, S. M. (2009). Intra-operative fluid warming in elective caesarean section: a blinded randomised controlled trial [Article]. International Journal of Obstetric Anesthesia, 18(4), 346-351.

Woolnough, M. J., Newton, R. S., Walters, M., & Chebbout, R. (2016). Active warming for elective caesarean section: a randomised controlled trial [Journal: Conference Abstract]. International Journal of Obstetric Anesthesia, 26, S10‐.

Xu, H., Luo, J., & Huang, F. (2007). [Clinical study on effect of keeping perioperative normal body temperature on skin flap survival] [Randomized Controlled Trial]. Chung-Kuo Hsiu Fu Chung Chien Wai Ko Tsa Chih/Chinese Journal of Reparative & Reconstructive Surgery, 21(7), 718-721.

Xu, H., Xu, G., Ren, C., Liu, L., Wei, L., & Schaller, B. (2019). Effect of forced-air warming system in prevention of postoperative hypothermia in elderly patients: A Prospective controlled trial. Medicine, 98(22), e15895-e15895.

Xu, L., Zhao, J., Huang, Y. G., & Luo, A. L. (2004). [The effect of intraoperative warming on patient core temperature] [Randomized Controlled Trial]. Chung-Hua Wai Ko Tsa Chih [Chinese Journal of Surgery], 42(16), 1010-1013.

Yam, P. C., & Carli, F. (1990). Maintenance of body temperature in elderly patients who have joint replacement surgery. A comparison between the heat and moisture exchanger and heated humidifier [Comparative Study; Journal Article]. Anaesthesia, 45(7), 563‐565.

Yamakage, M., Kawana, S., Yamauchi, M., Kohro, S., & Namiki, A. (1995). Evaluation of a forced-air warming system during spinal anesthesia. Journal of Anesthesia, 9(1), 93-95.

Yan, P., Mu, X. L., Zheng, W. D., Cui, J. F., Liu, J. H., Ma, Y. F., Gao, X., Hao, S. F., & Du, J. (2019). Design and manufacture of medical carbon fiber thermostatic heating pads [Article]. Chinese Journal of Tissue Engineering Research, 23(10), 1588-1593.

Yan, X., Rui, Z., Na, L., Chunmiao, H., Chunguang, R., Huiying, X., Xiao, Y., Zhang, R., Lv, N., Hou, C., Ren, C., & Xu, H. (2020). Effects of a preoperative forced-air warming system for patients undergoing video-assisted thoracic surgery: A randomized controlled trial. Medicine, 99(48), 1-9.

Yang, G., Zhu, Z., Zheng, H., He, S., Zhang, W., & Sun, Z. (2021). Effects of different thermal insulation methods on the nasopharyngeal temperature in patients undergoing laparoscopic hysterectomy: a prospective randomized controlled trial [Randomized Controlled Trial Research Support, Non-U.S. Gov't]. BMC Anesthesiology, 21(1), 101.

Yasar, P. O., Uzumcugil, F., Pamuk, A. G., & Kanbak, M. (2022). Comparison of Combined Forced-Air Warming and Circulating-Water-Mattress and Forced-Air Warming Alone in Patients Undergoing Open Abdominal Surgery in Lithotomy Position: a Randomized Controlled Trial. Indian Journal of Surgery, 84(1), 72-78.

Yeh, C. Y., Wang, M. S., Wang, W. J., Ho, K., Lin, T. S., Susetio, L., & Chao, C. C. (1989). Prevention of hypothermia during abdominal surgery: comparison of thermal tube and blanket [Clinical Trial Comparative Study Randomized Controlled Trial]. Ma Tsui Hsueh Tsa Chi Anaesthesiologica Sinica, 27(2), 153-156.

Yi, J., Liang, H., Song, R., Xia, H., & Huang, Y. (2018). Maintaining intraoperative normothermia reduces blood loss in patients undergoing major operations: a pilot randomized controlled clinical trial [Randomized Controlled Trial Research Support, Non-U.S. Gov't]. BMC Anesthesiology, 18(1), 126.

Yildirim, S., Unal, C. B., Dongel, I., Duger, C., Sahin, A. F., & Ersan, I. (2012). Effects of intraoperative skin surface warming on postanesthetic recovery and shivering: A prospective, randomized, clinical trial [Article]. HealthMED, 6(10), 3340-3345.

Yılmaz, H., & Khorshid, L. (2022). The Effects of Active Warming on Core Body Temperature and Thermal Comfort in Patients After Transurethral Resection of the Prostate: A Randomized Clinical Trial [Article in Press]. Clinical Nursing Research, 10547738221090593.

Yin, W., Wan, Q., Jia, H., Jiang, X., Luo, C., & Zhang, L. (2022). Comparison of two different uses of underbody forced-air warming blankets for the prevention of hypothermia in patients undergoing arthroscopic shoulder surgery: a prospective randomized study [Article]. BMC Anesthesiology, 22(1).

Yokoyama, K., Suzuki, M., Shimada, Y., Matsushima, T., Bito, H., & Sakamoto, A. (2009). Effect of administration of pre-warmed intravenous fluids on the frequency of hypothermia following spinal anesthesia for Cesarean delivery [Randomized Controlled Trial]. Journal of Clinical Anesthesia, 21(4), 242-248.

Yoo, H. S., Park, S. W., Yi, J. W., Kwon, M. I., & Rhee, Y. G. (2009). The effect of forced-air warming during arthroscopic shoulder surgery with general anesthesia [Comparative Study Randomized Controlled Trial]. Arthroscopy, 25(5), 510-514.

Yoo, J. B., Park, H. J., Chae, J. Y., Lee, E. J., Shin, Y. J., Ko, J. S., & Kim, N. C. (2013). Effects of ASPAN's evidence-based clinical practice guidelines for promotion of hypothermia of patients with total knee replacement arthroplasty [Article]. Journal of Korean Academy of Nursing, 43(3), 352-360.

Yoo, J. H., Ok, S. Y., Kim, S. H., Chung, J. W., Park, S. Y., Kim, M. G., Cho, H. B., Song, S. H., Cho, C. Y., & Oh, H. C. (2021). Efficacy of active forced air warming during induction of anesthesia to prevent inadvertent perioperative hypothermia in intraoperative warming patients: Comparison with passive warming, a randomized controlled trial [Randomized Controlled Trial]. Medicine, 100(12), e25235.

Yoo, J. H., Ok, S. Y., Kim, S. H., Chung, J. W., Park, S. Y., Kim, M. G., Cho, H. B., Song, S. H., Choi, Y. J., Kim, H. J., & Oh, H. C. (2022). Comparison of upper and lower body forced air blanket to prevent perioperative hypothermia in patients who underwent spinal surgery in prone position: a randomized controlled trial [Randomized Controlled Trial]. Korean Journal of Anesthesiology, 75(1), 37-46.

Yoshie, T., & Utsumi, I. (2012). The efficacy of prewarming on post-induction core temperature and thermoregulatory response under general anesthesia [Conference Abstract]. European Journal of Anaesthesiology, 29, 30.

Yu, Y., Wang, H., Wei, C., Wang, Z., Chen, Y., Wang, X., Quan, S., & Ni, X. (2017). Intra-operative warming blood transfusion contributes little to post-operative recovery quality in patients undergoing joint arthroplasty [Article]. International Journal of Clinical and Experimental Medicine, 10(2), 2487-2495.

Zaman, S. S., Rahmani, F., Majedi, M. A., Roshani, D., & Valiee, S. (2018). A Clinical Trial of the Effect of Warm Intravenous Fluids on Core Temperature and Shivering in Patients Undergoing Abdominal Surgery [Randomized Controlled Trial]. Journal of PeriAnesthesia Nursing, 33(5), 616-625.

Zeba, S., Surbatovic, M., Marjanovic, M., Jevdjic, J., Hajdukovic, Z., Karkalic, R., Jovanovic, D., & Radakovic, S. (2016). Efficacy of external warming in attenuation of hypothermia in surgical patients [Randomized Controlled Trial]. Vojnosanitetski Pregled, 73(6), 566-571.

Zhang, J., Deng, L., Wang, X., Song, F., Hou, H., & Qiu, Y. (2021). Effect of Forced-Air Warming Blanket on Perioperative Hypothermia in Elderly Patients Undergoing Laparoscopic Radical Resection of Colorectal Cancer [Article in Press]. Therapeutic Hypothermia and Temperature Management.

Zhao, J., Luo, A. L., Xu, L., & Huang, Y. G. (2005). Forced-air warming and fluid warming minimize core hypothermia during abdominal surgery [Randomized Controlled Trial]. Chinese Medical Sciences Journal, 20(4), 261-264.

Zhao, X. (2020). Effect of hypothermia prevention in patients undergoing gastrointestinal cancer surgery [Article]. International Journal of Clinical and Experimental Medicine, 13(10), 7638-7645.

Zheng, X., Sun, D., Zhou, F., & Zhang, Y. J. (2017). [Effects of perioperative thermoregulation on patients' body temperature, peripheral circulation and blood coagulation time in patients undergoing elective vertical hemi laryngectomy] [Randomized Controlled Trial]. Lin Chuang Er Bi Yan Hou Tou Jing Wai Ke Za Zhi = Journal Of Clinical Otorhinolaryngology, Head, & Neck Surgery, 31(14), 1113-1115.

محمود, ع., حسن محمدی پور, ا., علیرضا, ل., خسرو, ک., میر عباس, ه., & ، ندا, ب. (2020). تاثیر نرمال سالین وریدی گرم روی وضعیت همودینامیک و لرز بعد از جراحیهای گوش،حلق و بینی. Medical Journal of Tabriz University of Medical Sciences & Health Services, 42(2), 208-214.

范满祥, 叶玮, 徐小英, & 李霞. (2013). 综合保温措施对肠癌手术患者术后切口感染发生的影响 [Effect of perioperative heat preservation on surgical incision infections in colorectal cancer patients after colectomy]. 中华医院感染学杂志, 23(5), Article 2013376535.

冯斌. (2018). 手术室护理在腹腔镜直肠癌术中低体温护理的临床效果. 医学理论与实践, 31(24), Article 2019149023.

顾敏君, 孙浩, 许玉, 王鑫怡, 濮茜虹, & 李艳. (2021). 全麻下体温保护对老年胃肠手术患者凝血功能及血清乳酸含量的影响. 中外医学研究, 19(35), 5.

何盈盈, 郑慕华, & 张玉芳. (2018). 主动保温干预措施预防全身麻醉患者术中低体温的效果. Nursing of Integrated Traditional Chinese & Western Medicine, 4(6), 123-125.

黄宇, 尹东, 黄晓, 莫冰峰, 黄文文, & 石伟发. (2018). 复合保温措施对全髋关节置换术围手术期失血影响研究. 中国矫形外科杂志, 26(5), Article 2018300712.

金呀曼, 金楚珍, 林圣丹, & 张小秋. (2019). 不同保温护理干预对泌尿外术后低体温麻醉复苏期的干预效果分析. 重庆医学, 48(S2), Article 2020253454.

兰晓娥, 程伟琴, 王巧玲, 姚美蓉, 吴雪华, & 郭志华. (2012). 综合保温预防腹腔镜术中患者低体温的观察 [Effects of Comprehensive Warm Keeping Measures on Preventing Patients from Hypothermia in Laparoscopic Surgery]. 中华全科医学, 10(12), Article 2013203753.

李娜, 孙晓红, & 宋雪松. (2011). 复合保温对腹腔镜胆囊切除术患者体温及血流动力学的影响 [Effects of comprehensive heat preservation methods on body temperature and hemodynamics of patients undergoing laparoscopic cholecystectomy surgery]. 大连医科大学学报, 33(4), Article 2011768878.

李志华. (2012). 不同保温措施对胃癌根治术患者体温变化的影响. 河南外科学杂志, 18(5), Article 2012587340.

梁浩, & 易杰. (2017). 主动充气保温系统预防大手术患者术中低体温的效果 [Effectiveness of Underbody Forced-air Warming System in Preventing Hypothermia in Patients Undergoing Major Surgeries]. 中国医学科学院学报, 39(3), Article 2017484678.

刘欢. (2018). 术中下肢保温措施对老年粗隆间骨折术后患者深静脉血栓形成的影响 [Effects of intraoperative warming for lower limbs on deep venous thrombosis among elderly patients with intertrochanteric fracture surgery]. 中华现代护理杂志, 24(30), Article 2019183424.

刘剑. (2018). 手术中后期复合保温措施对颅脑手术患者低体温和不良反应的影响 [Effect of compound heat preservation in middle and late operation on hypothermia and adverse reaction of patients undergoing craniocerebral operation]. 中华现代护理杂志, 24(15), Article 2018386551.

刘淑凤, & 邵兵. (2019). 手术期间患者低体温的预防性护理措施的应用观察. 世界最新医学信息文摘(40), Article 2020205610.

刘松伟. (2017). 围术期体温护理在老年骨折手术患者中的应用效果分析. 中国伤残医学, 25(24), Article 2018195524.

刘晓艳, 郭鹏飞, 朱修源, 张瑾, 郑贵玲, & 李卉. (2019). 术中保温对开腹手术患者术后感染的影响 [Impact of intraoperative warming on postoperative infection in patients undergoing open surgery]. 中华医院感染学杂志, 29(33), Article 2020255467.

莫惠美. (2014). 保温护理对髋关节置换术术中低体温的影响. 国际护理学杂志(4), Article 2014408410.

覃文杰, 尹东, 黄宇, 黄晓, & 李富林. (2015). 全髋关节置换术中保温干预对围手术期出血的影响 [The effect of temperature interventions on perioperative blood loss in total hip replacement]. 中国矫形外科杂志, 23(17), Article 2015772960.

汪艳, 汪静, & 吴彦蒂. (2021). 术中静脉加温仪结合舒适干预对脑动脉瘤夹闭术患者的影响. 齐鲁护理杂志, 27(4), 3.

希仁古丽.亚森. (2018). 手术室护理干预对普外科腹腔镜手术患者低体温及其并发症的影响. 当代护士·学术版, 25(1), Article 2018171018.

向爱琳. (2016). 术中保温护理对肝癌患者低体温发生率及凝血功能的影响探讨 [Effect of Intraoperative Heat-preservation Nursing on the Incidence of Hypothermia and Blood Coagulation Function in Patients with Liver Cancer]. 中外医疗, 35(7), Article 2016340498.

薛颖, 路潞, & 樊雅静. (2021). 保温护理干预策略对妇科腔镜手术患者的影响. 齐鲁护理杂志, 27(2), 2.

叶陆游, 许心弦, & 潘孝云. (2018). 肩关节镜手术中灌洗液温度对患者体温及炎症反应的影响 [Effect of temperature of lavage fluid on body temperature and inflammation in arthroscopic shoulder surgery]. 中国骨与关节损伤杂志, 33(7), Article 2018459448.

박 효, 선., & 강 윤, 희. (2018). Effects of Heated-Humidified Anesthetic Gas in the Elderly Patients with Colorectal Cancer during Laparoscopic Surgery: Randomized Controlled Trial. Korean Journal of Adult Nursing, 30(2), 206-215. https://doi.org/10.7475/kjan.2018.30.2.206

张瑾. (2015). 保温护理对68例手术室患者术中应激影响的临床观察 [Clinical Observation of the Effect of Thermal Insulation on the Intraoperative Stress Reaction of 68 Patients in the Operating Room]. 中外医疗, 34(20), Article 2016172854.

张俊峰, & 仓静. (2009). 食管癌根治术中不同保温策略的效果及其对术后寒战的影响 [Outcomes of two temperature maintenance strategies during radical resection for carcinoma of oesophagus and their effects on postoperative shivering]. 上海交通大学学报·医学版, 29(6), Article 2009369932.

张献玲, 张政, 罗刚健, & 罗慧. (2017). 多模式体温干预措施对原位肝移植患者围手术期低体温的影响 [Effects of multi-modal temperature intervention measures on perioperative hypothermia in patients undergoing orthotopic liver transplantation]. 中华肝脏外科手术学电子杂志, 6(4), Article 2017569360.

张增欣, 范业琴, 陈淑华, 姜建军, 马诚芳, & 齐冰洁. (2011). 护理干预预防经皮肾镜取石术后低体温的效果 [Effect of nursing intervention on preventing hypothermia in patients with complicate renal calculi treated with percutaneous nephrolithotomy]. 中华现代护理杂志, 17(35), Article 2012264870.

赵香琴, 王凤, 余红, & 刘素蓉. (2018). "路径式"综合保温在妇科腔镜手术患者中应用的效果. 国际护理学杂志, 37(16), Article 2018563323.

周双峰. (2009). 术中输入加温液体对剖宫产孕妇寒颤的影响. 浙江临床医学, 11(6), Article 2009355302.

周学颖, 赵峰, 王萃, & 周乐. (2016). 复合保温对降低老年患者手术部位感染的效果分析 [Effect of composite temperature-keeping measures for reducing surgical site infections in elderly patients]. 中华医院感染学杂志, 26(5), Article 2016303624.

**References to ongoing studies**

ACTRN12617000850370. (2017). A randomised controlled trial investigating the effect of humidified warm carbon dioxide (CO2) insufflation during laparoscopic and open abdominal surgery.

ACTRN12619001570178. (2019). Efficacy of warm humidified insufflation for reducing post-operative ileus in patients undergoing acute general surgical laparotomy: A randomised single-blind controlled trial.

ACTRN12620001321932. (2020). Effect of air humidification and warming on surgical site temperature during spinal surgery.

ChiCTR-IPR-17011099. (2017). Clinical and Health Economic Benefits of Active Warming System.

ChiCTR1900022257. (2019). Aggressive vs. standard body temperature management on lung complications in elderly patients receiving curative resection of esophageal carcinoma: a randomized controlled trial.

ChiCTR2000033142. (2020a). Comparison of forced-air-warming blanket and intravenous fluid warming device on the prevention of intraoperative hypothermia in patients undergoing general surgery.

ChiCTR2000031845. (2020b). Comparison of the efficacy of three active warming methods during perioperative lumbar surgery of aged patients: a randomized controlled trial.

ChiCTR2000033756. (2020c). Comparison of the use of medical heating blankets and traditional body surface heating techniques and their postoperative complications in lumbar spine surgery: a prospective randomized controlled trial.

ChiCTR2000035207. (2020d). The effect of intraoperative target body temperature management on the incidence of emergency delirium: a randomized controlled trial.

ChiCTR2000033763. (2020e). A randomized controlled trial on the effect of integrated management mode of wireless temperature monitoring and intelligent warming for prevention intraoperative hypothermia in laparoscopic rectal cancer surgery.

ChiCTR2100047238. (2021). Influence of placement of inflatable heating blanket on body temperature and complications in patients undergoing elective open abdominal surgery.

CTRI/2020/02/023415. (2020). Comparison of effects of prewarming in patients of Hysterectomy for prevention of hypothermia.

CTRI/2021/04/033265. (2021). A clinical trial to study the effect of prewarming on intraoperative blood loss and transfusion requirements in Total Hip Arthroplasty surgeries.

Duff, J., Di Staso, R., Cobbe, K.-A., Draper, N., Tan, S., Halliday, E., Middleton, S., Lam, L., & Walker, K. (2012). Preventing hypothermia in elective arthroscopic shoulder surgery patients: a protocol for a randomised controlled trial. BMC Surgery, 12(1), 14-14.

Irct20190729044364N. (2019). The effect of electric blanket on post-cesarean shivering undergone spinal anesthesia in Bushehr's Persian Gulf hospital.

Irct20191224045885N. (2020). The effect of forced air warming and warmed intravenous fluid on comfort and prevention of shivering.

ISRCTN34213075. (2017). Warming perioperative multilayer blanket assessment.

JPRN-UMIN000020285. (2016a). Effect of hypothermia prevention during surgery by Warming before the general anesthesia introduction in the lung resection.

JPRN-UMIN000021761. (2016b). Relative clinical heat transfer effectiveness: Forced-air warming (Warm Touch TM) vs. Conductive fabric electric warming (HOT DOG TM).

JPRN-UMIN000028306. (2017a). Single center, exploratory research of pre- and intra-operative neck warming for the prevention of peri-operative hypothermia on laparoscopic abdominal surgery.

JPRN-UMIN000027991. (2017b). Usefulness of underbody-type blanket for forced-air warming in surgical patients under general anesthesia.

JPRN-UMIN000037256. (2019). The effect of head heat retention for the purpose of body temperature maintenance at the time of laparoscopic descent position surgery by general anesthesia-Method of optimum head heat retention maintenance.

JPRN-UMIN000040065. (2020). Effects of pre-warming during epidural combined general anesthesia on body temperature reduction by induction of anesthesia.

JPRN-UMIN000043699. (2021). Efficacy for Prevention of Perioperative Hypothermia by Warming Both Lower Extremities During Holmium Laser Enucleation of the Prostate.

KCT0005969. (2021). Effects of prewarming on preventing early intraoperative hypothermia during percutaneous nephrolithotomy under general anesthesia: a randomized controlled trial.

NCT03330067. (2017). Warm and Humidified vs Cold and Dry Carbon Dioxide (CO2) Pneumoperitoneum.

NCT03453866. (2018a). Effect of Warmed Irrigation in Hip Arthroscopy Undergoing Hip Arthroscopy.

NCT03429205. (2018b). The Efficacy of External Warming During Laparoscopic Bariatric Surgery.

NCT03581721. (2018c). Prevention of Maternal Hypothermia After Scheduled Caesarean Section Using Active Intravenous Warming.

NCT04187378. (2019a). Effect of Active Warming on Surgical Site Infections.

NCT03876808. (2019b). The Effect of Convective Pre-warming on Intra-operative Thermoregulatory Capabilities.

NCT04033900. (2019c). Effects of Active Prewarming in Perioperative Hypothermia in Adults.

NCT04011462. (2019d). Perioperative Normothermia: Temperature and Prewarming Methods.

NCT03878901. (2019e). Systemic Prevention and Management for Perioperative Hypothermia and Its Effect on Patients Outcome.

NCT04601636. (2020a). Comparison of Active Prewarming Versus Standard Care to Prevent Perioperative Hyporthermia in Short Outpatient Surgery Under General Anesthesia.

NCT04410068. (2020b). Comparison of Electric Heating Pad Versus Forced-air Warming to Prevent Inadvertent Perioperative Hypothermia.

NCT04667000. (2020c). The Effect of Forced Air Warming During Caseraen Section on Maternal Hypothermia: Randomized Controlled Trial.

NCT04991272. (2021a). 10 Minutes Prewarming and Warmed Intravenous Fluid on Core Temperature.

NCT04776954. (2021b). Comparison of Normothermia Maintenance Between Resistive Blanket and Forced Air Warming Systems in Renal Transplant Surgery.

NCT04907617. (2021c). The Effect of Active Warming During General Anaesthesia on Postoperative Body Temperature, Shivering and Thermal Comfort.

NCT04997694. (2021d). Effect of Preoperative Active Warming and Passive Warming Methods on Perioperative Hypothermia.

NCT05063292. (2021e). Effect of Prewarming on Skin Temperature Changes.

NCT04761224. (2021f). Impact of Intraoperative Instillation of Normothermal Saline on the Prevention of Intraoperative Hypothermia and Perioperative Morbidity of Prostatic Enucleation with Holmium Laser.

NCT04996407. (2021g). Survival Thermal Blanket Versus Draping Fabric to Prevent Hypothermia in Geriatric Surgical Patients.

NCT05314075. (2022a). Determination of Core Body Temperature in Parturient Warmed With Upper or Underbody Forced Air Cover (Bair Hugger).

NCT05213377. (2022b). Preoperative Warming, Hypothermia and Functional Recovery in Total Hip Arthroplasty.

Ryczek, E., White, J., Poole, R. L., Reeves, N. L., Torkington, J., & Carolan-Rees, G. (2019). Normothermic Insufflation to Prevent Perioperative Hypothermia and Improve Quality of Recovery in Elective Colectomy Patients: Protocol for a Randomized Controlled Trial. JMIR RESEARCH PROTOCOLS, 8(12).

TCTR20201008002. (2020). Comparing the effectiveness of force air warming to the upper or lower body to prevent hypothermia during abdominal surgery: Randomized controlled trial.

Zhang, J., Song, S., & Zhu, Q. (2021). Impact of multimodal warming during general anaesthesia on postoperative cognitive dysfunction in elderly patients with gynaecological cancer: study protocol for a single-blinded randomised controlled trial [Clinical Trial Protocol Research Support, Non-U.S. Gov't]. BMJ Open, 11(11), e049186.
