# Supplementary material for: Effect of active warming on perioperative cardiovascular outcomes: a systematic review and meta-analysis of randomized controlled trials
Source: J Anesth. 2023 Jun 8;37(4):573–81. doi: 10.1007/s00540-023-03205-4 (PMC10390383; doi:10.1007/s00540-023-03205-4)
Supplement: Supplementary file 3 — Supplementary file1 (DOCX 3479 KB) [file 540_2023_3205_MOESM3_ESM.docx]

**Effect of active warming on perioperative cardiovascular outcomes: a systematic review and meta-analysis of randomized controlled trials**

**Journal of Anesthesia**

Yunying Feng, Yuelun Zhang, Boyuan Sun, Yumiao He, Lijian Pei, Yuguang Huang^*^

*Correspondence to: Prof. Yuguang Huang, M.D., Department of Anesthesiology, Peking Union Medical College Hospital, Chinese Academy of Medical Sciences and Peking Union Medical College, 1 Shuaifuyuan, Dongcheng District, 100730 Beijing, China (email: [garypumch@163.com](mailto:garypumch@163.com)).

Supplementary Table 2. Characteristics of included studies.

| Publication information | Surgical type | Anaesthesia type | Body covered | Group sets | *n* | Perioperative cardiac complications | | | | | Duration of stay | | |
| --- | --- | --- | --- | --- | --- | --- | --- | --- | --- | --- | --- | --- | --- |
|  |  |  |  |  |  | All-cause mortality | MACE | MINS | Hypotension | Arrhythmia | PACU (min) | ICU (h) | Hospital  (day) |
| Kurz 1996 | Abdominal | General | Upper | No active warming | 96 | 2 | -- | -- | -- | -- | -- | -- | -- |
|  |  |  |  | i.v. fluids warming + Intra-operatvie forced-air warming | 104 | 2 | -- | -- | -- | -- | -- | -- | -- |
| Frank 1997 | Abdominal, thoracic, or vascular | General, Epidural, Combined | Upper or lower | i.v. fluids warming | 158 | 2 | 10 | -- | -- | 11 | -- | 22 (18-24)^a^ | 8 (5-13)^a^ |
|  |  |  |  | i.v. fluids warming + Intra-operative forced-air warming | 142 | 2 | 2 | -- | -- | 3 | -- | 21 (18- 23)^a^ | 8 (5-11)^a^ |
| Wong 2007 | Abdominal | General | FAW: Upper  WM: Below | i.v. fluids warming + Intra-operative forced-air warming | 56 | -- | 2 | -- | -- | -- | -- | -- | 9 (5-40)^a^ |
|  |  |  |  | i.v. fluids warming + Intra-operative forced-air warming + Warming mattress | 47 | -- | 0 | -- | -- | -- | -- | -- | 11 (5-119)^a^ |
| Kim 2014 | Knee arthroplasty | Spinal | FAW: Upper  WM: Below | i.v. fluids warming + Intra-operative forced-air warming | 23 | -- | -- | -- | -- | 0 | -- | -- | -- |
|  |  |  |  | i.v. fluids warming + Intra-operative circulating-water mattress | 23 | -- | -- | -- | -- | 2 | -- | -- | -- |
| Darvall 2016 | Neurosurgical | General | Full-length | No active warming | 15 | -- | -- | -- | 14 | -- | -- | -- | -- |
|  |  |  |  | Pre-operatvie forced-air warming 1h | 15 | -- | -- | -- | 15 | -- | -- | -- | -- |
| Schroeder 2016 | Hip arthroplasty | General | Above | Intra-operative forced-air warming (Bair Hugger) | 51 | -- | 0 | -- | -- | -- | -- | -- | -- |
|  |  |  |  | Pre-operative & intra-operative forced-air warming (Bair Paw) | 47 | -- | 1 | -- | -- | -- | -- | -- | -- |
| Chiang 2017 | Infra-inguinal | General | Above | i.v. fluids warming + Intra-operative forced-air warming | 18 | -- | 0 | -- | -- | -- | -- | -- | -- |
|  |  |  |  | i.v. fluids warming + Intra-operative forced-air warming + Warming mattress | 17 | -- | 1 | -- | -- | -- | -- | -- | -- |
| Unlügenç 2018 | Caesarean | Spinal | Above | Convective warming | 30 | -- | -- | -- | 21 | -- | -- | -- | -- |
|  |  |  |  | i.v. fluids warming + Convective warming | 30 | -- | -- | -- | 17 | -- | -- | -- | -- |
| Aydin 2019 | Lumbar stabilization | General | Below | No active warming | 30 | -- | -- | -- | 7 | 0 | -- | -- | -- |
|  |  |  |  | i.v. fluids warming + Intra-operative forced-air warming | 30 | -- | -- | -- | 3 | 1 | -- | -- | -- |
| Canturk 2019 | Caesarean | Spinal | -- | No active warming | 30 | -- | -- | -- | 23 | 2 | -- | -- | -- |
|  |  |  |  | i.v. fluids warming | 30 | -- | -- | -- | 22 | 3 | -- | -- | -- |
| Zhang 2019 | Esophageal cancer | General | Lower | i.v. fluids warming | 35 | -- | -- | 11 | -- | 10 | -- | 65.8 (37.3-112.5)^b^ | 13 (10-18)^b^ |
|  |  |  |  | i.v. fluids warming + Intra-operative forced-air warming | 35 | -- | -- | 3 | -- | 1 | -- | 66.1 (36.1-88.5)^b^ | 13 (11-15)^b^ |
| Akelma 2020 | Prostatic transurethral | General | Above | No active warming | 17 | -- | -- | -- | 9 | -- | 45.64 (17.11)^c^ | -- | -- |
|  |  |  |  | Pre-operatvie 30min & post-operative forced-air warming | 16 | -- | -- | -- | 1 | -- | 31.18 (9.57)^c^ | -- | -- |
| Ni 2020 | Caesarean | Spinal | Upper | No active warming | 64 | -- | -- | -- | 28 | -- | -- | -- | -- |
|  |  |  |  | i.v. fluids warming + Pre-operatvie forced-air warming 30min | 68 | -- | -- | -- | 30 | -- | -- | -- | -- |
| Hu 2021 | Abdominal | General | Upper | No active warming | 37 | -- | -- | -- | -- | 1 | -- | -- | -- |
|  |  |  |  | Intra-operatvie forced-air warming | 37 | -- | -- | -- | -- | 0 | -- | -- | -- |
| Becerra 2021 | Bladder or prostatic transurethral | General | Full-length | Intra-operative forced-air warming | 76 | -- | 1 | -- | -- | 1 | 156 (72)^c^ | -- | -- |
|  |  |  |  | Pre-operative 15min & Intra-operative forced-air warming | 74 | -- | 0 | -- | -- | 0 | 122 (58)^c^ | -- | -- |
|  |  |  |  | Pre-operative 30min & Intra-operative forced-air warming | 73 | -- | 0 | -- | -- | 0 | 114 (56)^c^ | -- | -- |
|  |  |  |  | Pre-operative 45min & Intra-operative forced-air warming | 74 | -- | 0 | -- | -- | 0 | 118 (64)^c^ | -- | -- |
| Breuer 2022 | Gynecologic laparoscopic | General | FAW: Above | i.v. fluids warming + Cold & dry insufflation gas+ Intra-operative forced-air warming | 49 | -- | -- | -- | 1 | 31 | 88 (27-318)^d^ | -- | 4.5 (1-10)^d^ |
|  |  |  |  | i.v. fluids warming + Warm & humidified insufflation gas | 50 | -- | -- | -- | 5 | 35 | 95 (10-270)^d^ | -- | 4.5 (0.5-14)^d^ |
|  |  |  |  | i.v. fluids warming + Warm & humidified insufflation gas + Intra-operative forced-air warming | 49 | -- | -- | -- | 2 | 34 | 90 (30-235)^d^ | -- | 4.75 (1-13.5)^d^ |
| Ji 2022 | Thoracoscopic | General | Lower | No active warming | 49 | -- | -- | -- | -- | 5 | 40.1 (3.7)^c^ | -- | -- |
|  |  |  |  | i.v. fluids warming + Intra-operatvie forced-air warming | 49 | -- | -- | -- | -- | 1 | 37.1 (4.3)^c^ | -- | -- |
| Sessler 2022 | Orthopaedic, laparoscopic, open abdominal, neurosurgical, urological, other | General | Upper or lower | No active warming | 2506 | 17 | 60 | 223 | 2 | 10 | -- | -- | 8 (6-12)^b^ |
|  |  |  |  | i.v. fluids warming + Pre-operative 30 min & intra-operatvie forced-air warming | 2507 | 13 | 57 | 233 | 5 | 3 | -- | -- | 8 (6-12)^b^ |
| Wang 2022 | Laparoscopic | General | Full-length | i.v. fluids warming + Intra-operatvie forced-air warming (38℃) | 63 | -- | -- | -- | 14 | 10 | -- | -- | -- |
|  |  |  |  | i.v. fluids warming + Intra-operatvie forced-air warming (42℃) | 64 | -- | -- | -- | 20 | 10 | -- | -- | -- |
|  |  |  |  | i.v. fluids warming + Intra-operatvie forced-air warming (42℃ for 1h, then 38℃) | 64 | -- | -- | -- | 20 | 15 | -- | -- | -- |

^a^ Values are presented as mean (range).

^b^ Values are presented as median (interquartile range).

^c^ Values are presented as mean (standard deviation).

^d^ Values are presented as median (range).

FAW, forced-air warming; ICU, intensive care unit; i.v. fluids, intravenous fluids; MACE, major adverse cardiac events; MINS, myocardial injury after noncardiac surgery; PACU, post-anaesthesia care unit; WM, warming mattress.

Supplementary Table 3. Characteristics of the included studies in final meta-analysis.

| Publication information | Surgical type | Anaesthesia type | Body covered | Group sets | *n* | Mean core temperature (°C)^a^ | Perioperative cardiac complications | | | | |
| --- | --- | --- | --- | --- | --- | --- | --- | --- | --- | --- | --- |
|  |  |  |  |  |  |  | All-cause mortality | MACE | MINS | Hypotension | Arrhythmia |
| Kurz 1996 | Abdominal | General | Upper | No active warming | 96 | 34.7 (0.6) | 2 | -- | -- | -- | -- |
|  |  |  |  | i.v. fluids warming + Intra-operatvie forced-air warming | 104 | 36.6 (0.5) | 2 | -- | -- | -- | -- |
| Frank 1997 | Abdominal, thoracic, or vascular | General, Epidural, Combined | Upper or lower | i.v. fluids warming | 158 | 35.4 (0.1) | 2 | 10 | -- | -- | 11 |
|  |  |  |  | i.v. fluids warming + Intra-operative forced-air warming | 142 | 36.7 (0.1) | 2 | 2 | -- | -- | 3 |
| Darvall 2016 | Neurosurgical | General | Full-length | No active warming | 15 | 36.2 (0.2) | -- | -- | -- | 14 | -- |
|  |  |  |  | Pre-operatvie forced-air warming 1h | 15 | 36.8 (0.4) | -- | -- | -- | 15 | -- |
| Aydin 2019 | Lumbar stabilization | General | Below | No active warming | 30 | 36.3 (0.2) | -- | -- | -- | 7 | 0 |
|  |  |  |  | i.v. fluids warming + Intra-operative forced-air warming | 30 | 36.5 (0.2) | -- | -- | -- | 3 | 1 |
| Zhang 2019 | Esophageal cancer | General | Lower | i.v. fluids warming | 35 | 35.80 (0.18) | -- | -- | 11 | -- | 10 |
|  |  |  |  | i.v. fluids warming + Intra-operative forced-air warming | 35 | 36.61 (0.18) | -- | -- | 3 | -- | 1 |
| Akelma 2020 | Prostatic transurethral | General | Above | No active warming | 17 | 36.18 (0.35) | -- | -- | -- | 9 | -- |
|  |  |  |  | Pre-operatvie 30min & post-operative forced-air warming | 16 | 36.03 (0.33) | -- | -- | -- | 1 | -- |
| Hu 2021 | Abdominal | General | Upper | No active warming | 37 | 36.32 (0.20) | -- | -- | -- | -- | 1 |
|  |  |  |  | Intra-operatvie forced-air warming | 37 | 36.79 (0.06) | -- | -- | -- | -- | 0 |
| Ji 2022 | Thoracoscopic | General | Lower | No active warming | 49 | 36.20 (0.19) | -- | -- | -- | -- | 5 |
|  |  |  |  | i.v. fluids warming + Intra-operatvie forced-air warming | 49 | 36.49 (0.20) | -- | -- | -- | -- | 1 |
| Sessler 2022 | Orthopaedic, laparoscopic, open abdominal, neurosurgical, urological, other | General | Upper or lower | No active warming | 2506 | 35.6 ± 0.3 | 17 | 60 | 223 | 2 | 10 |
|  |  |  |  | i.v. fluids warming + Pre-operative 30 min & intra-operatvie forced-air warming | 2507 | 37.1 ± 0.3 | 13 | 57 | 233 | 5 | 3 |

^a^ The mean core temperatures are the core temperature measured at the end of surgery. Values are presented as mean (standard deviation).

Supplementary Figure 1. Risk of bias summary: review authors’ judgements about each domain for each included study according to the Cochrane Collaboration’s tool for assessing risk of bias.


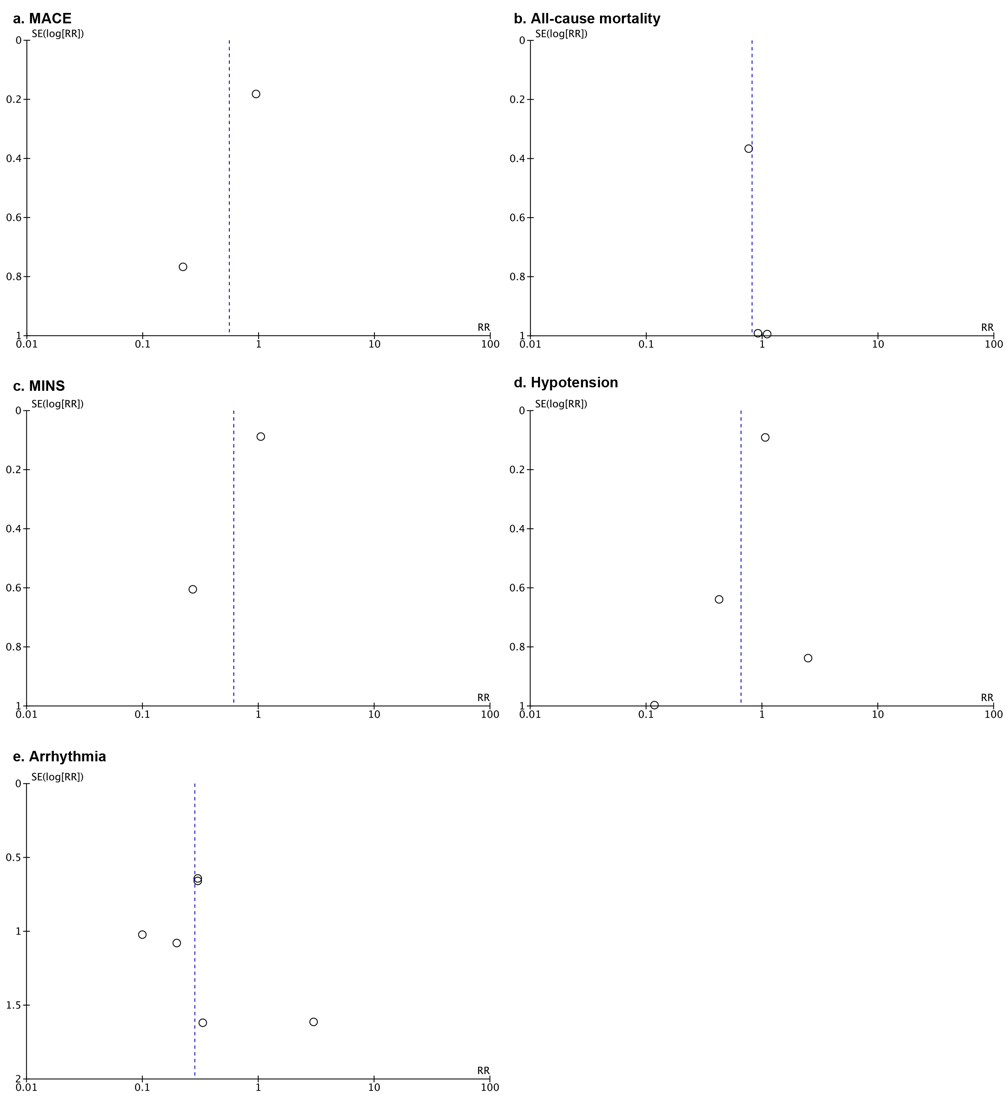
Supplementary Figure 2. Funnel plots.
